# Design, Synthesis, Biological Evaluation, and Molecular Modeling of 2-Difluoromethylbenzimidazole Derivatives as Potential PI3Kα Inhibitors

**DOI:** 10.3390/molecules27020387

**Published:** 2022-01-08

**Authors:** Xiangcong Wang, Moxuan Zhang, Ranran Zhu, Zhongshan Wu, Fanhong Wu, Zhonghua Wang, Yanyan Yu

**Affiliations:** 1 School of Chemical and Environmental Engineering, Shanghai Institute of Technology, 100 Haiquan Road, Shanghai 201400, China; wxcwayne@126.com (X.W.); kmjln1261545@163.com (M.Z.); zrr206062216@126.com (R.Z.); wzsjy2020@163.com (Z.W.); wfh@sit.edu.cn (F.W.); 2 Shanghai Engineering Research Center of Green Fluoropharmaceutical Technology, 100 Haiquan Road, Shanghai 201400, China

**Keywords:** PI3Kα inhibitor, 3D-QSAR, molecular docking, molecular dynamics simulation, benzimidazoles, anticancer

## Abstract

PI3Kα is one of the potential targets for novel anticancer drugs. In this study, a series of 2-difluoromethylbenzimidazole derivatives were studied based on the combination of molecular modeling techniques 3D-QSAR, molecular docking, and molecular dynamics. The results showed that the best comparative molecular field analysis (CoMFA) model had q^2^ = 0.797 and r^2^ = 0.996 and the best comparative molecular similarity indices analysis (CoMSIA) model had q^2^ = 0.567 and r^2^ = 0.960. It was indicated that these 3D-QSAR models have good verification and excellent prediction capabilities. The binding mode of the compound **29** and 4YKN was explored using molecular docking and a molecular dynamics simulation. Ultimately, five new PI3Kα inhibitors were designed and screened by these models. Then, two of them (**86**, **87**) were selected to be synthesized and biologically evaluated, with a satisfying result (22.8 nM for **86** and 33.6 nM for **87**).

## 1. Introduction

PI3K/AKT/mTOR signaling is a therapeutic targeting pathway for the development of cancer treatment, which is activated aberrantly in many human cancers [1,2,3].

Phosphoinositide 3-kinase (PI3K) is a kind of signal transducing enzyme that mediates key cellular functions in cancer and immunity and could be categorized into three classes (class I, class II, and class III) based on sequence homology [4,5,6,7]. Class I PI3Ks are further classified into class IA (PI3Kα, β, and δ) and class IB (PI3Kγ), which have been extensively studied [1,8,9]. Their main function is to catalyze the 3-hydroxyl phosphorylation of the phosphatidylinositol 4,5-diphosphate (PIP2) inositol ring to phosphatidylinositol 3,4,5-triphosphate (PIP3), which facilitates the activation of its downstream effectors [10,11,12].

Class II PI3Ks are composed of PI3KC2α PI3KC2β, and PI3KC2γ, which modulate intracellular membrane dynamics and membrane traffic [13]. Class III PI3Ks have as the only member VPS34, which participates in vesicle trafficking and autophagy [13].

Among the subtypes of PI3K, PI3Kα is most often associated with cancer and controls a variety of physiological processes as an important regulator of intracellular signaling pathways [8,14]. The aberrant activation of PI3Kα and its downstream effectors is related to tumor initiation and maintenance [15]. The selective targeting of the PI3Kα isoform has special pharmacological significance, which makes PI3Kα an attractive target in anticancer drug development [10,16]. However, although great efforts have been made to explore PI3Kα inhibitors, there are few reports on PI3Kα inhibitors that possess the ability to differentiate between PI3Kα and its isoforms [17].

**Figure 1:**
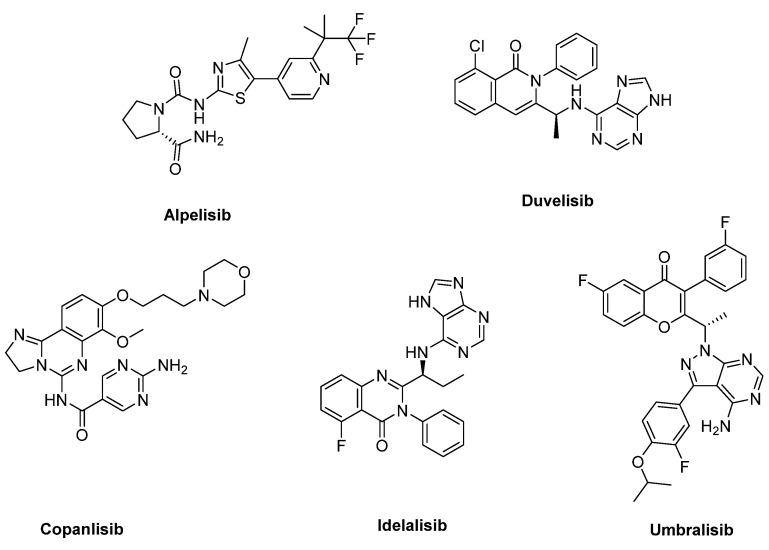
Structures of the PI3K inhibitors approved by the FDA.

Many potent PI3K inhibitors have been disclosed since the discovery of the first-generation inhibitors, for example, Wortmannin and LY294002 [18]. Five of them (Idelalisib, Copanlisib, Duvelisib, Alpelisib, and Umbralisib) have been approved by the FDA, and the corresponding structures are depicted in Figure 1. It is worth noting that Alpelisib is a PI3Kα-specific inhibitor and was approved by the Food and Drug Administration (FDA) in 2019. It is used in combination with fulvestrant to treat HR+/HER2− advanced breast cancer patients [19]. It is assumed that the selective PI3Kα inhibitors could protect the patients from the side effects associated with a broader inhibition, which allows greater suppression of the signaling pathway and durable antitumor efficacy [7].

In this study, 3D-QSAR models were developed using CoMFA [20] and CoMSIA [21] technology based on 85 2-difluoromethylbenzimidazole derivatives [22,23,24]. The relationships between the key structural and pharmacodynamic information were obtained from the 3D-QSAR model, which were helpful for further drug research [25,26,27,28,29,30]. The binding mode of the compound **29** with 4YKN was explored through molecular docking and molecular dynamics simulation. Ultimately, five new PI3Kα inhibitors were designed and evaluated. Two of them were selected to be synthesized and biologically evaluated. Compound **86** (IC_50_ = 22.8 nM) and compound **87** (IC_50_ = 33.6 nM) showed potent inhibitory activity against PI3Kα.

## 2. Results and Discussion

### 2.1. CoMFA and CoMSIA Statistical Results

To verify the predictive ability of the model, all key statistical parameters were analyzed. The statistical parameters of standard CoMFA and CoMSIA models are given in Table 1. For CoMFA analysis, Partial Least Square (PLS) analysis showed cross-validated correlation coefficient (q^2^) value of 0.797, non-cross-validated correlation coefficient (r^2^) of 0.996, and the optimal number of components (ONC) of 10 in the training set. The contribution of the electrostatic field (50.4%) to the binding affinity was close to that of the steric field (49.6%). For CoMSIA analysis, PLS analysis showed q^2^ value of 0.567, r^2^ of 0.960, Fisher test (F) = 230.278, and Standard Error of Estimate (SEE) of 0.099, with 6 components in the training set.

### 2.2. 3D-QSAR Contour Map Results and Analysis

**Figure 2:**
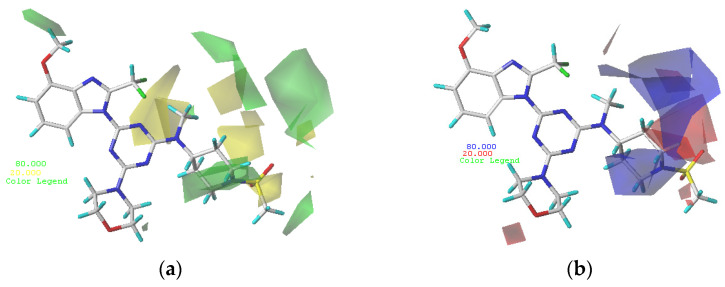
Contour maps of compound **29**. (**a**) Steric contour maps of CoMFA: favored (green) and disfavored (yellow); (**b**) electrostatic contour maps of CoMFA: electrostatic (blue) and electronegative (red).

**Figure 3:**
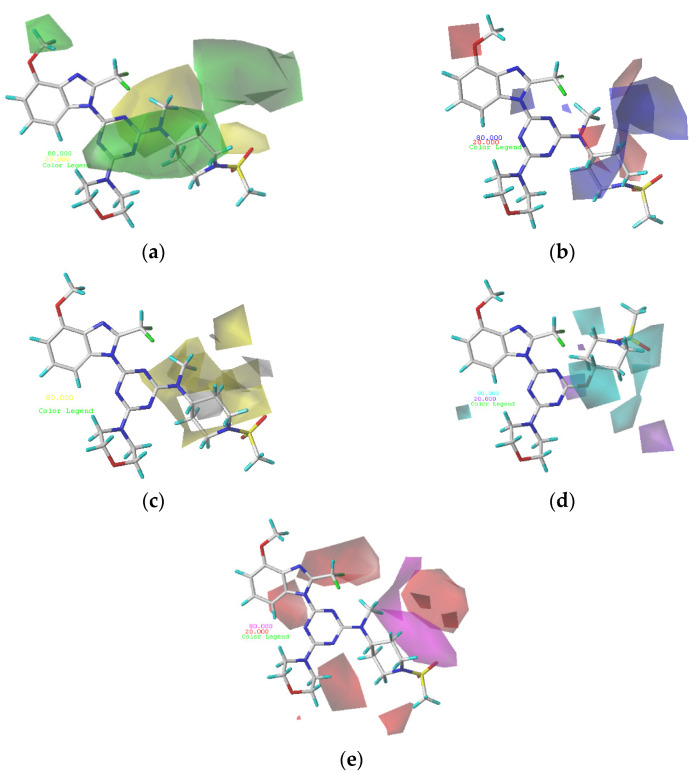
(**a**) Steric contour maps of CoMSIA; (**b**) electrostatic contour maps of CoMSIA; (**c**) hydrophobic contour maps of CoMSIA: favored (yellow) and disfavored (gray); (**d**) hydrogen bond donor contour maps of CoMSIA: favored (cyan) and disfavored (purple); (**e**) hydrogen bond acceptor contour maps of CoMSIA: favored (magenta) and disfavored (red).

In the optimized CoMFA and CoMSIA models, the most active compound, **29**, was used as a template molecule in all subsequent contour maps (Figure 2 and Figure 3). Contour maps are important for designing new drug candidates because they show areas where changes in the molecular field energy are closely related to changes in biological activity.

The steric and electrostatic fields from the optimal CoMFA model are shown in Figure 2. As shown in the steric map (Figure 2a), areas where steric bulk substituents increased the potency are represented by green polyhedrons, while areas where steric bulk substituents decreased the potency are represented by yellow polyhedrons. The medium green contour occurring at R_2_ of compound **29** indicates that compounds with bulk substituents at this site would have better biological activity. Compound **2** (pIC_50_ = 8.4), with a methoxy group at R_2_, possesses better biological activity than compound **1** (pIC_50_ = 8.05), without methoxy substitution. The medium-yellow contour in the near-methylsulfonyl group at R_1_ of compound **29** indicates that compounds with small substitutions would have better biological activity. Compound **5** (pIC_50_ = 8.22), with the methylsulfonyl group, possesses better biological activity than compound **6** (pIC_50_ = 7.68), with the ethylsulfonyl group. A large green contour occurring in the piperidine ring at R_1_ of compound **29** indicates that compounds with bulk substituents at this site would have better biological activity. Compound **17** (pIC_50_ = 8.29), with a 6-membered ring, possesses better biological activity than the corresponding compound **21** (pIC_50_ = 8.12), with a 5-membered ring, and compound **25** (pIC_50_ = 7.49), with a 4-membered ring.

As shown in the electrostatic map (Figure 2b), the blue area indicates that the electropositive group is beneficial to biological activity and the red area indicates that the electronegative group is beneficial to biological activity. A large blue contour occurring near the 2′-site of the piperidine ring at R_1_ of compound **29** indicates that compounds with electropositive substituents at this site would have better biological activity. Compound **29** (pIC_50_ = 8.64), with -N at the 1′-site of the piperidine ring, possesses better biological activity than the corresponding compound **31** (pIC_50_ = 7.7), with -N at the 2′-site of the piperidine ring. A small red contour occurring above the aminomethyl group of R_1_ of compound **29** indicates that compounds with electronegative substituents at this site would have better biological activity. Compound **29** (pIC_50_ = 8.64), with the aminomethyl group, possesses better biological activity than compound **28** (pIC_50_ = 7.85), with the imino group.

Figure 3a depicts the CoMSIA steric contour map of the optimal model with compound **29** overlaid. In this map, the green contours (sterically beneficial) and yellow contours (sterically unbeneficial) contours represent 80% and 20% level contributions, respectively. A small green contour occurring above R_2_ of compound **29** indicates that compounds with bulk substituents at this site would have better biological activity. Compound **5** (pIC_50_ = 8.22), with the methoxy group at R_2_, possesses better biological activity than compound **4** (pIC_50_ = 7.68), without the methoxy group. A medium-yellow contour occurring near the sulfonamide group indicates that compounds with small substituents at this site would have better biological activity. Compound **47** (pIC_50_ = 7.11), with -NH_2_ not in the yellow contour, possesses slightly better biological activity than the corresponding compound **48** (pIC_50_ = 6.97), with -NH_2_ in the yellow contour.

The electrostatic contour map of the CoMSIA model is displayed in Figure 3b. The blue (electropositive group favorable) and red (electronegative group favorable) contours represent 80% and 20% level contributions, respectively. A medium-red contour occurring near the oxygen atom of R_1_ of compound **29** indicates that compounds with electronegative substituents at this site would have better biological activity. Compound **2** (pIC_50_ = 8.64) with -OCH_3_ possess better biological activity than compound **1** (pIC_50_ = 7.85), with -H. A medium-blue contour was observed on the piperidine ring of R_1_, which revealed that an improvement in the electropositivity of this site would improve the biological activity.

The CoMSIA contour map of hydrophobic contribution is described in Figure 3c. In this figure, the yellow (hydrophobic favorable) and white (hydrophobic unfavorable) contours represent 80% and 20% level contributions, respectively. Small yellow contours occurring near the 2′-site of piperidin at R_1_ of compound **29** indicates that the hydrophobic group at this site would improve the biological activity. Compound **10** (pIC_50_ = 8.49), with -CH_2_ at the 4′-site of piperidin, possesses better biological activity than compound **9** (pIC_50_ = 8.13), without -CH_2_. Small white contours covering the 2′,3′-site of piperidin at R_1_ of compound **29** indicates that a hydrophobic group at this site would reduce the biological activity.

In Figure 3d, the cyan and purple contour maps indicate favorable and unfavorable H-bond donor groups, representing 80% and 20% level contributions, respectively. A medium cyan contour occurring at R_1_ illustrates that compounds with an H-bond donor substituent at this site were preferred to have higher PI3Kα inhibitory activity. Compound **3** (pIC_50_ = 8.51), with -NH_2_, possesses slightly better biological activity than compound **2** (pIC_50_ = 8.40), with -H. A medium purple contour is located blew the piperidine ring at R_1_ of compound **29**, indicating the H-bond donor substituents that had bad biological activity.

As shown in Figure 3e, the hydrogen bond acceptor field of the CoMSIA model is represented by magenta (hydrogen bond acceptor favorable) and red (hydrogen bond acceptor unfavorable), representing 80% and 20% level contributions, respectively. Small red contours of the methylsulfonyl group suggest that the introduction of hydrogen bond acceptor substituents to these regions would decrease biological activity. A medium magenta contour near the piperidin ring at R_1_ of compound **29** shows that hydrogen bond acceptor substituents would have better biological activity. Compound **7** (pIC_50_ = 8.12), with -N(CH_3_)_2_, possesses better biological activity than compound **6** (pIC_50_ = 7.68), with -CH_2_CH_3_ in the yellow contour.

**Figure 4:**
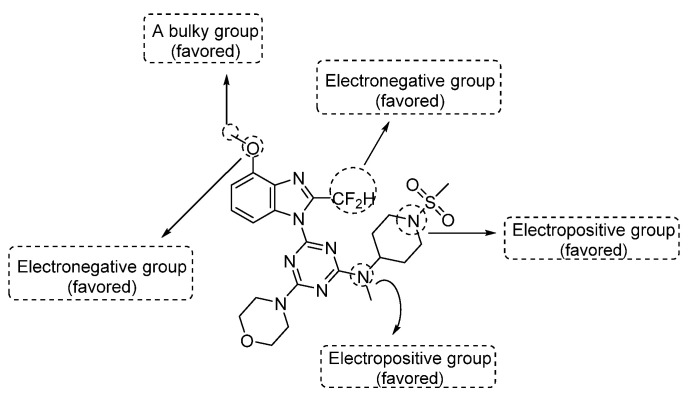
Structural modification tips derived from 3D-QSAR studies.

The above-mentioned contour analyses of CoMFA and CoMSIA are summarized in Figure 4, which provides effective help for the future design of new PI3Kα inhibitors.

### 2.3. Molecular Docking Results and Analysis

**Figure 5:**
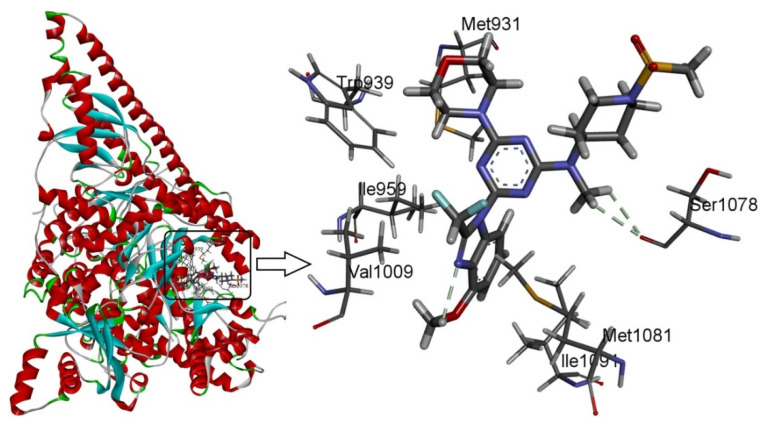
Docking of the representative ligand compound **29** into the binding site of PI3Kα. Ligands and the important residues of the binding interaction are depicted by stick-and-line models.

Docking studies were performed on compound **29** as the template. As shown in Figure 5, template compound **29** was docked in the binding cavity of the 4ykn with three non-classical H-bonds. An intramolecular H-bond (-H…N-, 2.62 Å) was built between -H of the methoxy group at R_2_ and the -N group at the 2′-site of the nuclear structure. Two hydrogen atoms of -NCH_3_ at R_1_ interacted through H-bonding with -O=C of Ser1078 (-H…O=C-, 2.60 Å, -H…O=C-, 2.65 Å), which increased the affinity between compound **29** and the protein.

### 2.4. Molecular Dynamics Simulation Results and Analysis

**Figure 6:**
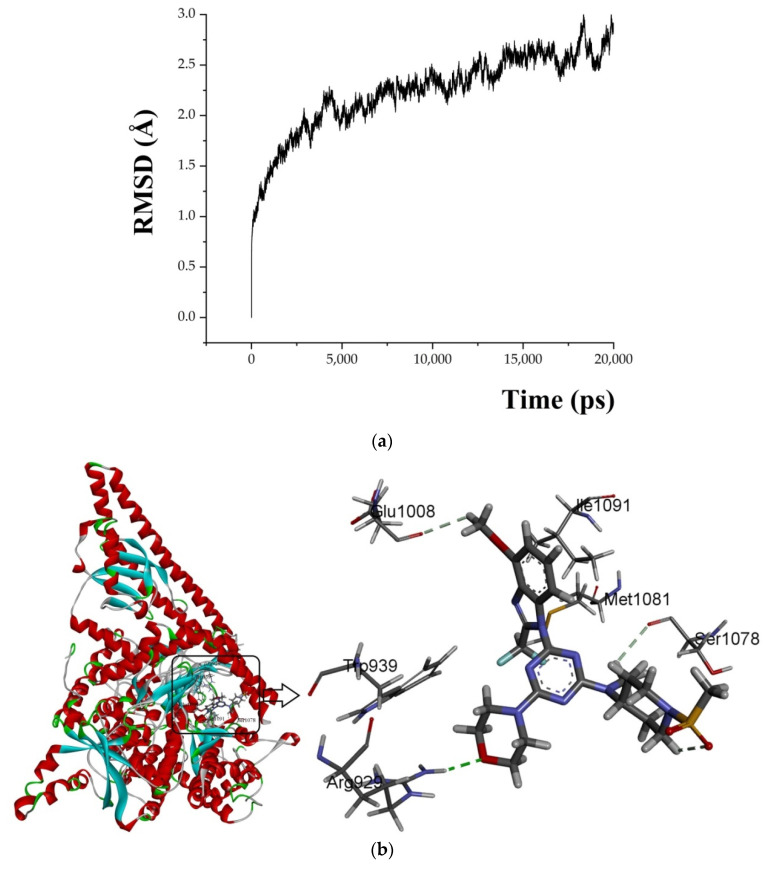

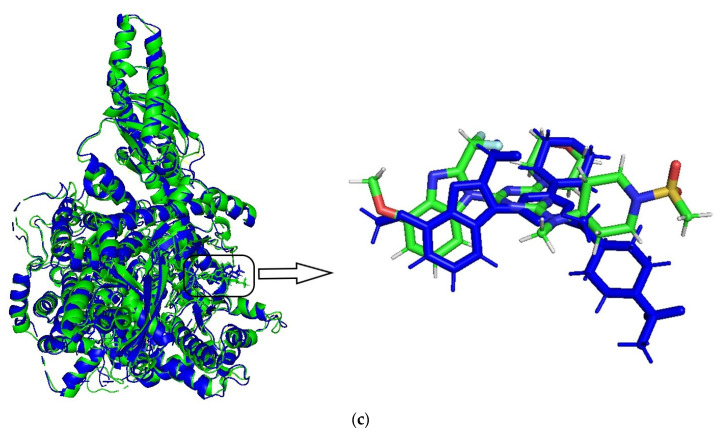
MD simulation results. (**a**) plot of the RMSD of the docked complex versus the MD simulation time in the MD-simulated structures; (**b**) Plot of the MD-simulated structure of the binding site with the ligand. Compound **29** was in the active site of PI3Kα. H-bonds are shown as dotted green lines; active site amino acid residues are represented as sticks; the inhibitors are shown as sticks; (**c**) view of superimposed backbone atoms of the lowest energy structure of the MD simulation (blue) and the initial structure (green) for the compound **29**–PI3Kα complex. Compound **29** is represented as a carbon chain in green for the initial complex and a carbon chain in blue for the lowest energy complex.

A 20 ns molecular dynamics (MD) simulation was carried out using the above-mentioned docking complexes (PI3Kα with ligand **29**). The main purpose of the simulation was to optimize the binding pocket and the correlation between PI3Kα and compound **29**. The root-mean-square deviation (RMSD) plots are shown in Figure 6a for the receptor (4YKN) and the ligand (compound **29**) to explore conformational variations. After 10 ns, the RMSD of the complex was about 2.5 Å, and it retained this value throughout the simulation. A superposition of the most stable structure in all MD simulations and the initial docked structure is shown in Figure 6b,c. After MD simulation, the interactions between compound **29** and the receptor were analyzed here in order to explore the similarity and difference between the results of docking and MD simulation. From the most stable structure extracted from the MD simulation, the model also revealed that one classical hydrogen bond and three non-classical hydrogen bonds are formed between the ligand and the receptor (shown in Figure 6a). The oxygen atom of the morpholine group at R_1_ interacts through classical H-bonding with -NH of Arg929 (-O…H-, 2.53 Å). The oxygen atom of methanesulfonyl at R_1_ interacts through intramolecular H-bonding with -H of the piperidine ring (-O…H-, 2.51 Å). The methoxy group at R_2_ forms a non-classical H-bond with Glu1008 (-C=O…H-, 2.76 Å). The hydrogen atom of -NCH_3_ at R_1_ interacts through a non-classical H-bond with -O=C of Ser1078 (-H…O=C-, 3.07 Å). The number of hydrogen bonds in this MD simulation result was more than that in the docking model, and a classic hydrogen bond was formed between the protein and the receptor. Therefore, the conformation obtained after MD simulation was more stable than the docked conformation. MD simulation is performed in a more realistic environment and much closer to physiological conditions.

### 2.5. Design and Prediction

**Figure 7:**
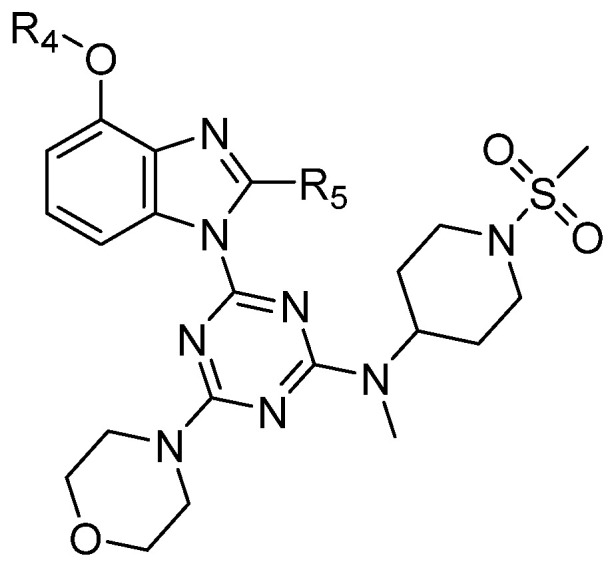
The structure of the designed compounds based on the template compound **29**.

On the basis of the above-mentioned analysis, five novel structures (compounds **86–90**), with -CH_3_/-CH_2_CH_3_/-C(CH_3_)_3/_CH_2_CH (CH_3_)CH_3_ for R_4_ and -CF_3_/-CHF_2_/-CHFBr for R_5_, were designed based on the template compound **29** (Figure 7). The predicted values are shown in Table 2. Compounds **86**–**90** might have higher activities than the template compound **29** in the 3D-QSAR models; it was indicated that these new designed compounds might become potential candidates as PI3Kα inhibitors. Compounds **86** and **87** were synthesized to validate the reliability of this 3D-QSAR model.

**Scheme 1:**
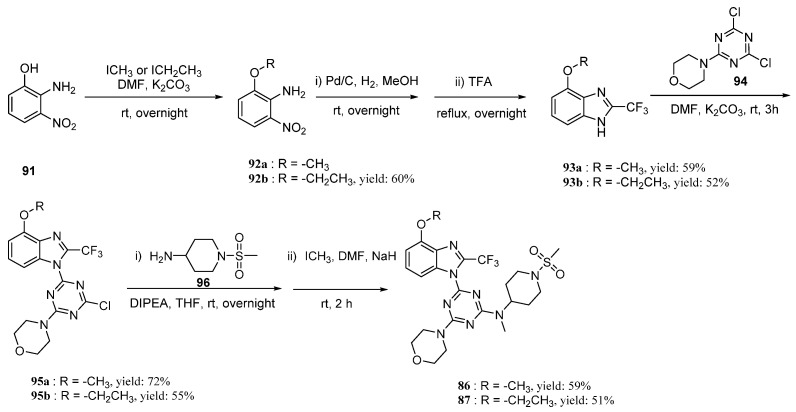
Synthesis of compounds **86** and **87**.

Based on the MD simulations, the binding free energy calculations for compounds **29**, **86**, and **87** of the last 2 ns trajectory were carried out. The binding free energy values for the compounds **29**, **86**, and **87** via MM/GBSA were calculated to be −23.83, −29.79, and −41.29 kcal/mol. This further shows that **86** and **87** would have better activity. Their synthesis route is shown in Scheme 1.

### 2.6. The Synthesis of Compounds ***86*** and ***87***

Two novel 2-difluoromethylbenzimidazole derivatives (**86**, **87**) were synthesized according to Scheme 1. The compounds **91**, **92a**, **94**, and **96** are commercially available and were used without further purification. The compounds **86b** and **87a** were synthesized with reference to previous literature [31,32,33]. Compounds **93a** or **93b** were synthesized by Pd/C hydrogenation and subsequent dehydration cyclization with trifluoroacetic acid, with moderate yields of 59% and 60%, respectively. Then, under the action of potassium carbonate in DMF, **95a** and **95b** were obtained via the first S_N_Ar reaction and the yields were 59% and 52%, respectively. Finally, after the second S_N_Ar reaction at the residual chlorine atom in **95a** and **95b** under slightly stronger reaction conditions, and subsequent methylation by methyl iodide, the desired compounds **86** and **87** were achieved with 59% and 51% yields, respectively.

### 2.7. Biological Evaluation

**Figure 8:**
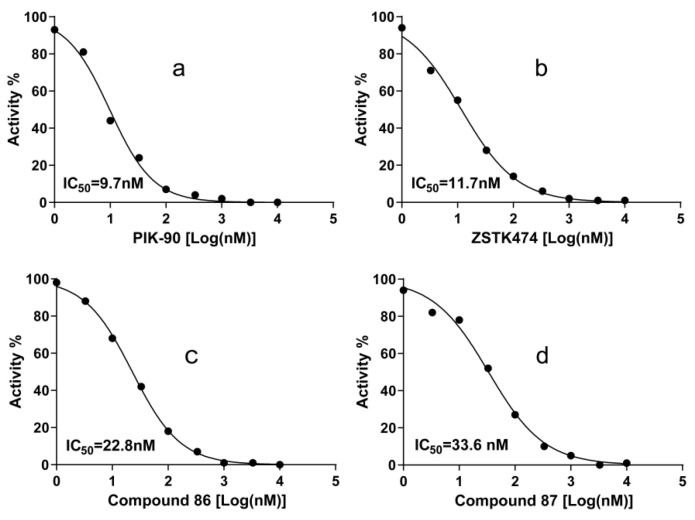
Dose response curves of PI3Kα inhibitors PIK-90 (**a**), ZSTK474 (**b**), compound **86** (**c**) and Compound **87** (**d**). IC_50_ values were measured with ATP as the PI3Kα substrate. Each experiment was carried out in duplicate and repeated three times.

IC_50_ values of the compound **86**, **87**, PIK-90, and ZSTK474 against PI3Kα are listed in Table 3 and Figure 8. ZSTK474 and PIK-90 were used as the reference drug. The results showed that compound **86** and **87** both have potent inhibitory activity against PI3Kα, close to that of ZSTK474.

## 3. Materials and Methods

### 3.1. Datasets and Biological Activity

In 3D-QSAR studies, a set of 85 2-difluoromethylbenzimidazole derivatives had good biological activity against PI3Kα, and these derivatives were collected from the literature [22,23,24]. All derivatives were allocated to the training set and the test set: the training set, containing 65 compounds (76%), would generate a 3D-QSAR model, and the test set, containing 20 compounds (24%), would confirm the reliability of the models. The test set was selected randomly so that the data set would show high structural diversity and a wide range of activities.

The CoMFA and CoMSIA models were created using the Sybyl X 2.0 software package, in which Gasteiger–Hückel [34] charge was added to all compounds, and its energy was minimized at the Tripos force field [35] using the Powell gradient algorithm. To obtain the most stable conformation, the convergence criterion is 0.005 kcal/mol·Å, and the maximum number of iterations was set to 10,000. Their chemical structures and active values against PI3Kα are listed in Supplementary Information (Table S1).

### 3.2. Molecular Modeling and Alignment

**Figure 9:**
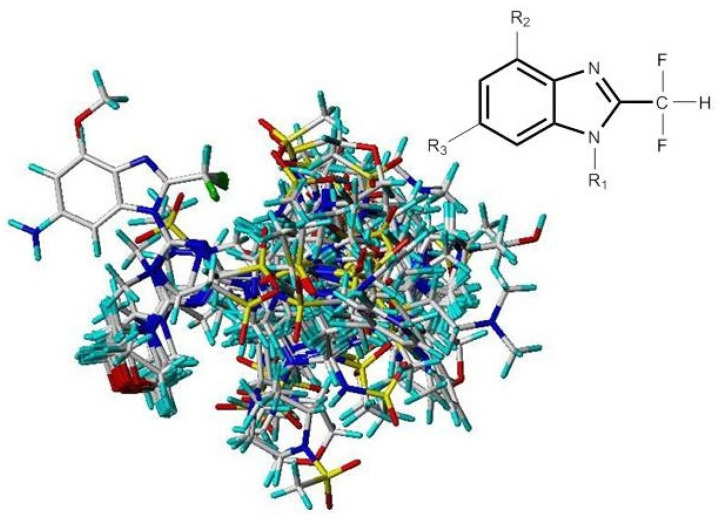
Molecular alignments of all compounds in the data set.

Molecules of the training sets were aligned onto the molecules that had the most potent inhibitor (compound **29**). Compound **29** was employed as the template molecule because of its highest PI3Kα inhibitory activity amongst training sets, and the corresponding results are shown in Figure 9. The common substructure is depicted in bold.

### 3.3. 3D-QSAR Models Studies

**Figure 10:**
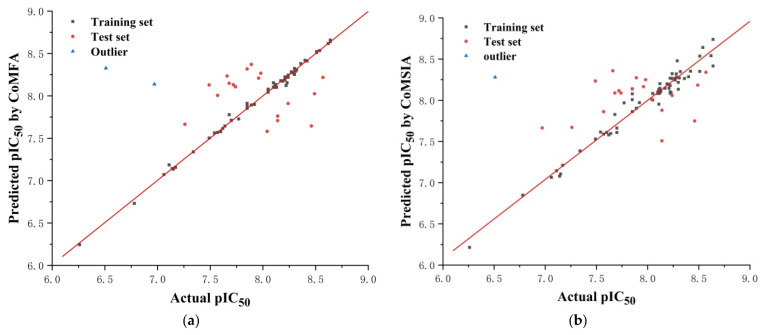
The correlation plots of the predicted pIC_50_ values versus the actual pIC_50_ values using the training set and the test set based on the CoMFA (**a**) model and the CoMSIA (**b**) model, respectively.

The reliability and predictive ability of the 3D-QSAR models were evaluated through internal and external validation. In the partial least squares (PLS) analysis, the leave-one-out (LOO) method was used to determine the optimum number of components (NOC). In the 3D-QSAR model validation, non-cross-validated correlation coefficient (r^2^_ncv_), standard error estimate (SEE), and F-statistic values (F) were obtained. Each molecule’s predicted value and actual value are in a linear relationship and, respectively, shown in Figure 10. The predicted pIC_50_ values via CoMFA and CoMSIA models are listed in Supplementary Information (Table S2).

Five fields (steric, electrostatic, donor, acceptor, and hydrophobic) were considered during PLS analysis. The relationship between actual and predicted pIC_50_ values of the training and test set molecules is illustrated in Figure 10.

Before this validation, an initial inspection of the predicted activities revealed poor predictions for compounds **14** and **48**, which were considered outliers in our work for both the ligand-based and receptor-based models. The steric hindrance of the morpholine ring of compound **14** was increased by two methylene bridges. The structure was different from that of other compounds, which was inconsistent with the model, and it led to abnormal activity values. The terminal methyl group of compound **48** was surrounded by a blue area in the electrostatic map, which was inconsistent with the model, and it resulted in abnormal activity values.

### 3.4. Molecular Docking

Molecular docking studies were carried out using the Surflex-Dock module in Sybyl-x 2.0, which aimed to analyze the detailed binding mode of small molecules and PI3Kα and to validate the 3D-QSAR models. The 3D structures of PI3Kα (PDB code: 4ykn) were downloaded from the RCSB protein database. The protein ligands and water were removed, and the pocket used to combine with molecules was exposed before docking.

### 3.5. Molecular Dynamics Simulation

A 20 ns molecular dynamics simulation was carried out on the docking complex to assess the reliability of the docking results and further explore the **29**—PI3Kα interactions. Amber 12.0 software [36] was used for molecular dynamics studies. The general AMBER force field (GAFF) and the Amber ff12SB were employed on the ligands and the proteins, respectively. Na^+^ was included to neutralize the system within a cubic water box. The entire molecular dynamics simulation followed the procedures for minimization, heating, density balance, and production. The steepest descent and the conjugate gradient method were used to minimize the energy of the whole system. The minimized system was heated to 300 K gradually over 20 ps under the NVT ensemble. Then 20 ns unrestrained MD simulations were performed in the NPT ensemble at 1 atm and a temperature of 300 K. All hydrogen atoms were constrained by SHAKE algorithm [37]. After the MD simulation, the root-mean-square deviation (RMSD) was calculated to evaluate the stability of the complex system.

### 3.6. Chemistry

#### 3.6.1. *2-Ethoxy-6-nitroaniline* **92b**

To a stirred solution of 2-amino-3-nitrophenol (5.000 g, 32.44 mmol) in DMF (25 mL) was added powdered K_2_CO_3_ (4.930 g, 35.69 mmol) and iodoethane (5.570 g, 35.69 mmol). The mixture was stirred at room temperature overnight and then diluted with NH_4_Cl (saturated) and extracted with EtOAc (×3). Organic layers were washed with water and brine, dried over Na_2_SO_4_, filtered, and concentrated. Purification by column chromatography (PE/EA = 4:1) through silica gel afforded the intermediate **92b** (3.560 g; 60%), a brown solid; the yield was: 60%. ^1^H-NMR (501 MHz, chloroform-*d*) δ 7.70 (dd, *J* = 8.9, 1.3 Hz, 1H), 6.85 (dd, *J* = 7.8, 1.4 Hz, 1H), 6.57 (dd, *J* = 8.9, 7.7 Hz, 1H), 6.44 (s, 2H), 4.10 (q, *J* = 7.0 Hz, 2H), 1.48 (t, *J* = 7.0 Hz, 3H).

#### 3.6.2. *4-Methoxy-2-(trifluoromethyl)-1H-benzo[d]imidazole* **93a**

To a stirred solution of **92a** (3.000 g, 17.84 mmol) in MeOH (40 mL) was added 10% Pd-C (0.869 g). After stirring at room temperature overnight under a hydrogen atmosphere, the reaction mixture was filtered through a pad of Celite. The filtrate was concentrated in vacuo to give the intermediate diamine, which was then dissolved in TFA (75 mL) initially at 0 °C. The resulting mixture was subsequently stirred overnight under reflux conditions before concentration to a residue in vacuo. The residue was dissolved in EtOAc, and the resulting solution was poured into saturated NaHCO_3_ aq. The organic layer was separated, dried over Na_2_SO_4_, and concentrated in vacuo to give **93a** (2.291 g), a white solid; the yield was: 59%. ^1^H-NMR (501 MHz, DMSO-*d*_6_) δ 14.05 (d, *J* = 126.4 Hz, 1H), 7.27 (d, *J* = 7.7 Hz, 2H), 6.97–6.65 (m, 1H), 3.94 (s, 3H).

#### 3.6.3. *4-Ethoxy-2-(trifluoromethyl)-1H-benzo[d]imidazole* **93b**

To a stirred solution of **92b** (3.560 g, 19.54 mmol) in MeOH was added 10% Pd-C (0.775 g). After stirring at room temperature overnight under a hydrogen atmosphere, the reaction mixture was filtered through a pad of Celite. The filtrate was concentrated in vacuo to give the intermediate diamine (3.260 g, 24.42 mmol), which was then dissolved in TFA (75 mL) initially at 0 °C. The resulting mixture was subsequently stirred overnight under reflux conditions before concentration to a residue in vacuo. The residue was dissolved in EtOAc and the resulting solution was poured into saturated NaHCO_3_ aq. The organic layer was separated, dried over Na_2_SO_4_, and concentrated in vacuo to give **93b** (2.300 g, 52% yield), a light-yellow liquid; the yield was: 52%. mp 198.3–198.4 °C. ^1^H-NMR (400 MHz, DMSO-*d*_6_) δ 13.99 (d, *J* = 91.1 Hz, 1H), 7.46–7.07 (m, 2H), 6.96–6.66 (m, 1H), 4.21 (q, *J* = 7.0 Hz, 2H), 1.39 (t, *J* = 7.0 Hz, 3H). ^13^C-NMR (101 MHz, DMSO-*d*_6_) δ 151.62, 136.09, 133.01, 126.42, 119.59 (q, *J* = 270.2 Hz), 105.19 (d, *J* = 27.1 Hz), 64.27, 15.06.^19^F-NMR (376 MHz, DMSO-*d*_6_) δ −62.56 (d, *J* = 82.1 Hz). HRMS (EI-TOF) calculated for C_10_H_9_F_3_N_2_O [M + H]^+^: 231.0739; found: 231.0739.

#### 3.6.4. *4-(4-Chloro-6-(4-methoxy-2-(trifluoromethyl)-1H-benzo[d]imidazol-1-yl)-1,3,5-triazin-2-yl)morpholine* **95a**

A mixture of **93a** (0.500 g, 2.31 mmol), 4-(4,6-dichloro-1,3,5-triazin-2-yl)morpholine (**94**) (2.170 g, 9.25 mmol), and powdered K_2_CO_3_ (2.560 g, 18.50 mmol) in DMF (20 mL) was stirred rapidly for 3 h and then diluted with water. The resulting precipitate was collected by filtration, washed with water and then with EtOH, and oven-dried to give **95a** (0.700 g), a white solid; the yield was: 72%. ^1^H-NMR (501 MHz, chloroform-*d*) δ 7.90 (d, *J* = 8.4 Hz, 1H), 7.42 (t, *J* = 8.3 Hz, 1H), 6.84 (d, *J* = 8.1 Hz, 1H), 4.04 (s, 3H), 3.94 (dt, *J* = 14.1, 4.9 Hz, 4H), 3.81–3.75 (m, 4H). ^13^C-NMR (126 MHz, chloroform-*d*) δ 171.04, 164.37, 161.75, 152.34, 139.80–138.57 (m), 135.19, 131.33, 128.42, 118.66 (q, *J* = 270.8 Hz), 108.33, 105.68, 66.46, 66.42, 55.99, 44.59, 44.48. ^19^F-NMR (376 MHz, chloroform-*d*) δ −60.12. HRMS (EI-TOF) calculated for C_16_H_14_ClF_3_N_6_O_2_ [M + H]^+^: 415.0891; found: 415.0892.

#### 3.6.5. *4-(4-Chloro-6-(4-ethoxy-2-(trifluoromethyl)-1H-benzo[d]imidazol-1-yl)-1,3,5-triazin-2-yl)morpholine* **95b**

A mixture of **93b** (1.000 g, 4.34 mmol), 4-(4,6-dichloro-1,3,5-triazin-2-yl)morpholine (94) (4.080 g, 17.38 mmol), and powdered K_2_CO_3_ (4.800 g, 34.75 mmol) in DMF (30 mL) was stirred rapidly for 3 h and then diluted with water. The resulting precipitate was collected by filtration, washed with water and then with EtOH, and oven-dried to give **95b** (1.026 g), a light-yellow liquid; the yield was: 55%. mp 230.1–230.5 °C. ^1^H-NMR (400 MHz, chloroform-*d*) δ 7.90 (d, *J* = 8.4 Hz, 1H), 7.41 (t, *J* = 8.3 Hz, 1H), 6.85 (d, *J* = 8.1 Hz, 1H), 4.33 (q, *J* = 7.0 Hz, 2H), 3.95 (dt, *J* = 10.2, 4.8 Hz, 4H), 3.83–3.75 (m, 4H), 1.55 (t, *J* = 7.0 Hz, 3H). ^13^C-NMR (101 MHz, chloroform-*d*) δ 171.13, 164.49, 161.88, 151.80, 135.42, 131.56, 128.43, 122.31–115.39 (m), 108.09, 106.83, 66.52, 66.48, 64.65, 44.65, 44.54, 14.74. ^19F^-NMR (376 MHz, chloroform-*d*) δ −60.00. (s, 3F) HRMS (EI-TOF) calculated for C_17_H_16_ClF_3_N_6_O_2_ [M + H]^+^: 429.1048; found: 429.1053.

#### 3.6.6. *4-(4-Methoxy-2-(trifluoromethyl)-1H-benzo[d]imidazol-1-yl)-N-methyl-N-(1-(methylsulfonyl)piperidin-4-yl)-6-morpholino-1,3,5-triazin-2-amine* **86**

A mixture of **95a** (0.050 g, 120.55 μmol), 1-(methylsulfonyl)piperidin-4-amine (0.085 g, 482.19 μmol), and DIPEA (0.023 g, 182.82 μmol) in THF (6 mL) was stirred at room temperature overnight. Dilution with water and extraction with CH_2_Cl_2_ gave an intermediate. A solution of the intermediate (0.036 g, 5.4 mmol) in DMF (10 mL) was treated sequentially with excess NaH (0.006 g, 258.73 μmol) and iodomethane (0.036 g, 258.73 μmol) at room temperature for 2 h. Dilution with water and workup in CH_2_Cl_2_, followed by chromatography on silica eluting with CH_2_Cl_2_/EtOAc (4:1), gave **86** (0.022 g), a light-yellow liquid; the yield was: 59%. ^1^H-NMR (400 MHz, DMSO-*d*_6_) δ 7.75 (dd, *J* = 34.1, 8.4 Hz, 1H), 7.48 (q, *J* = 8.5 Hz, 1H), 6.98 (d, *J* = 8.1 Hz, 1H), 4.64 (q, *J* = 11.8, 10.6 Hz, 1H), 3.98 (s, 3H), 3.87–3.64 (m, 10H), 3.06 (d, *J* = 5.9 Hz, 3H), 2.92 (d, *J* = 2.2 Hz, 3H), 2.90–2.69 (m, 2H), 1.93–1.68 (m, 4H). ^13^C-NMR (101 MHz, DMSO-*d*_6_) δ 165.19, 164.89, 161.28, 152.47, 135.73, 131.22, 128.51, 107.78, 106.06, 66.34, 56.36, 52.25, 45.72, 44.09, 35.11, 29.03, 28.46. ^19^F-NMR (376 MHz, DMSO-*d*_6_) δ −59.03 (d, *J* = 20.0 Hz). HRMS (EI-TOF) calculated for C_23_H_29_F_3_N_8_O_4_S [M + Na]^+^: 593.1876; found: 593.1880.

#### 3.6.7. *4-(4-Ethoxy-2-(trifluoromethyl)-1H-benzo[d]imidazol-1-yl)-N-methyl-N-(1-(methylsulfonyl)piperidin-4-yl)-6-morpholino-1,3,5-triazin-2-amine* **87**

A mixture of **95b** (0.200 g, 466.42 μmol), 1-(methylsulfonyl)piperidin-4-amine (0.332 g, 1.87 mmol), and DIPEA (0.090 g, 699.63 μmol) in THF (13 mL) was stirred at room temperature overnight. Dilution with water and extraction with CH_2_Cl_2_, followed by chromatography on silica eluting with CH_2_Cl_2_/EtOAc (4:1), gave an intermediate (0.198 g, 74% yield): 1H-NMR (CDCl_3_). A solution of the intermediate (0.198 g, 347.01 μmol) in DMF (10 mL) was treated sequentially with excess NaH (0.033g, 1.39 mmol) and iodomethane (0.197 g, 1.39 mmol) at room temperature for 2 h. Dilution with water and workup in CH_2_Cl_2_, followed by chromatography on silica eluting with CH_2_Cl_2_/EtOAc (4:1), gave **87** (0.102 g, 51% yield), a light-yellow liquid; the yield was: 51%. ^1^H-NMR (400 MHz, DMSO-*d*_6_) δ 7.72 (dd, *J* = 34.0, 8.4 Hz, 1H), 7.42 (dt, *J* = 10.9, 8.2 Hz, 1H), 6.92 (dd, *J* = 8.2, 2.8 Hz, 1H), 4.64 (tq, *J* = 16.7, 7.3, 5.6 Hz, 1H), 4.25 (qd, *J* = 7.0, 2.5 Hz, 2H), 3.88–3.59 (m, 10H), 3.03 (d, *J* = 7.2 Hz, 3H), 2.91 (s, 3H), 2.89–2.65 (m, 2H), 1.95–1.67 (m, 4H), 1.43 (t, *J* = 6.9 Hz, 3H). ^13^C-NMR (101 MHz, DMSO-*d*_6_) δ 165.19, 164.89, 161.35, 151.69, 135.81, 131.30, 128.44, 119.30, 107.62, 106.81, 66.35, 64.57, 55.39, 52.45, 52.02, 45.71, 44.08, 35.11, 29.03, 28.46, 15.11. ^19^F-NMR (376 MHz, DMSO-*d*_6_) δ −59.04 (s, 3F). HRMS (EI-TOF) calculated for C_24_H_31_F_3_N_8_O_4_S [M + Na]^+^: 607.2033; found: 607.2028.

### 3.7. Enzyme Assays

These assays were carried out as described previously [38]. All of the enzymatic reactions were conducted at 30 °C for 40 min. The 50 µL reaction mixture contained 40 mM Tris, pH 7.4, 10 mM MgCl_2_, 0.1 mg/mL BSA, 1 mM DTT, 10 µM ATP, kinase, and the enzyme substrate. The compounds were diluted in 10% DMSO, and 5 µL of the dilution was added to a 50 µL reaction so that the final concentration of DMSO is 1% in all of reactions. The assay was performed using the Kinase-Glo Plus luminescence kinase assay kit. It measured the kinase activity by quantitating the amount of ATP remaining in solution following a kinase reaction. The luminescent signal from the assay was correlated with the amount of ATP present and was inversely correlated with the amount of kinase activity. The IC_50_ values were calculated using nonlinear regression with normalized dose–response fit using Scikit-learn 1.0.2 [39].

## 4. Conclusions

In this work, the binding models between 2-difluoromethylbenzimidazole derivatives and PI3Kα were studied by a combination of 3D-QSAR, molecular docking, and molecular dynamics simulations. The contour maps explained the relationship between chemical structures and bioactivities. The molecular docking and molecular dynamics results implied that relevant hydrogen bonds are important for ligand–receptor binding. The results from the 3D-QSAR models, molecular docking, and MD simulation illustrated the chemical structure characteristics of 2-difluoromethylbenzimidazole derivatives. Ultimately, two designed compounds (**86** and **87**) were selected to be synthesized and biologically evaluated. Compound **86** (IC_50_ = 22.8 nM) and compound **87** (IC_50_ = 33.6 nM) show potent inhibitory activity against PI3Kα. This study provides guidance for the further development of potent PI3Kα inhibitors with improved biological activity.

## Figures and Tables

**Table 1:** The best results of the CoMFA and CoMSIA PLS statistical results.

Statistical Parameters	CoMFA	CoMSIA
q^2^	0.797	0.567
r^2^	0.996	0.960
ONC	10	6
SEE	0.032	0.099
F	1394.03	230.278
Field Contribution (%)		
Steric (S)	49.6	12.9
Electrostatic (E)	50.4	20.4
Hydrophobic (H)		19.4
Acceptor (A)		18.8
Donor (D)		28.4

**Table 2:** Structures of the compounds with predicted activity.

Compound	Structure	CoMFA	CoMSIA
		Predicted	Predicted
**86**	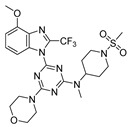	8.512	8.437
**87**	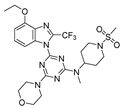	8.526	8.445
**88**	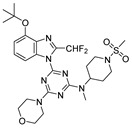	8.681	8.473
**89**	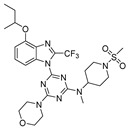	8.510	8.435
**90**	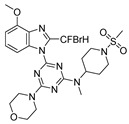	8.443	8.427

**Table 3:** IC_50_ values of compounds **86**, **87**, PIK-90, and ZSTK474 against PI3Kα.

Compound	IC_50_ (nM)
**86**	22.8 ± 0.4
**87**	33.6 ± 2.7
ZSTK474	11.7 ± 2.2
PIK-90	9.7 ± 1.1

## Data Availability

Data are contained within the article or the supplementary data file.

## Supplementary Materials

The following supporting information can be downloaded online, Table S1: Structures and activity value of every molecule used in the study, Table S2: The actual and predicted pIC_50_ values of all compounds, Figures S1–S22 ^1^H-NMR, ^19^F-NMR, ^13^C-NMR and HRMS spectra for the new drug.

## Sample Availability

Samples of the compounds are available from the authors.
