# Supplementary material for: Design, Synthesis, Biological Evaluation, and Molecular Modeling of 2-Difluoromethylbenzimidazole Derivatives as Potential PI3Kα Inhibitors
Source: Molecules. 2022 Jan 8;27(2):387. doi: 10.3390/molecules27020387 (PMC8777764; doi:10.3390/molecules27020387)
Supplement: Supplementary file 1 [file molecules-27-00387-s001.zip › molecules-1532155-supplementary.pdf]

Supplementary Information

Design, Synthesis, Biological Evaluation, and Molecular Modeling of 2-Difluoromethylbenzimidazole Derivatives as Potential PI3Kα Inhibitors

Xiangcong Wang [1], Moxuan Zhang [1], Ranran Zhu [1], Zhongshan Wu [1], Fanhong Wu [1,2], Zhonghua Wang [1,2,*] , and Yanyan Yu [1,2,*]

[1] School of Chemical and Environmental Engineering, Shanghai Institute of Technology, 100 Haiquan Road, Shanghai 201400, China; wxcwayne@126.com (X.W.); kmjln1261545@163.com (M.Z.); zrr206062216@126.com (R.Z.); wzsjy2020@163.com (Z.W.); wfh@sit.edu.cn (F.W.)

[2] Shanghai Engineering Research Center of Green Fluoropharmaceutical Technology, 100 Haiquan Road, Shanghai 201400, China

* Correspondence: wzhsit@163.com (Z.W.); sshnhyyy@sit.edu.cn (Y.Y.)

Table of contents

S2-S11: Table S1. Structures and activity value of every molecule used in the study

S11-S13: Table S2. The actual and predicted pIC_{50} values of all compounds.

S13-S24: ^{1}H NMR, ^{19}F NMR, ^{13}C NMR and HRMS spectra of the important compounds

Table S1. Structures and activity value of every molecule used in the study.

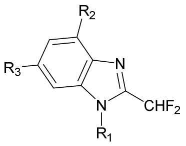

Compd.	Substituent			IC50 (nM)
	R1	R2	R3	
**1**	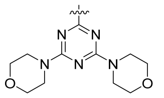	-H	-H	8.90
**2**	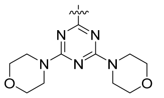	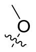	-H	4.00
**3**	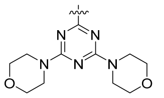	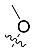	H_2N-	3.10
**4**	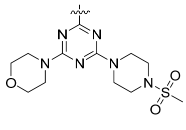	-H	-H	21.00
**5**	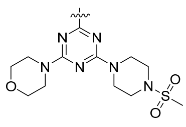	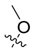	-H	6.00
**6**[TS]	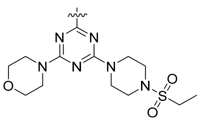	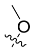	-H	21.00
**7**	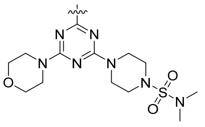	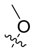	-H	7.60

**8**^{TS}	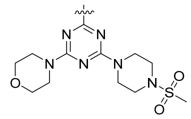	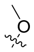	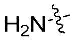	22.00
**9**	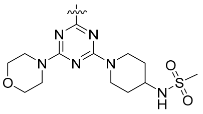	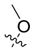	-H	7.40
**10**^{TS}	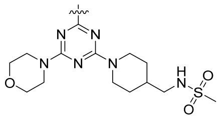	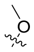	-H	3.20
**11**^{TS}	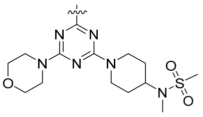	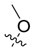	-H	14.00
**12**	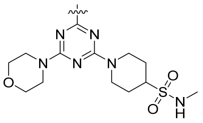	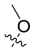	-H	14.00
**13**	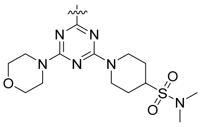	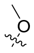	-H	17.00
***14**^{TS}	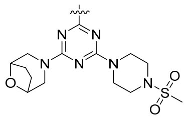	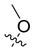	-H	306.00
**15**	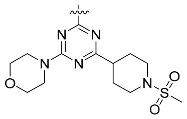	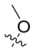	-H	5.30
**16**	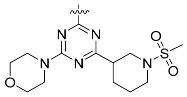	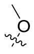	-H	6.70

**17**	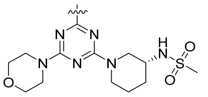	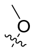	-H	5.10
**18**	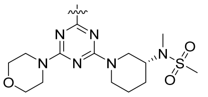	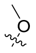	-H	2.90
**19**[TS]	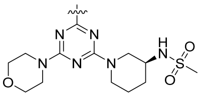	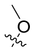	-H	10.50
**20**[TS]	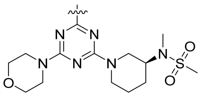	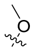	-H	19.00
**21**	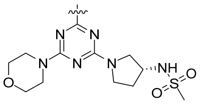	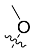	-H	7.60
**22**	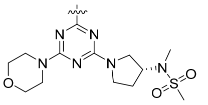	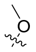	-H	3.10
**23**	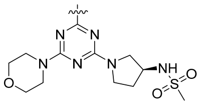	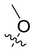	-H	4.80
**24**	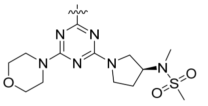	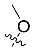	-H	7.70
**25**[TS]	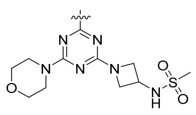	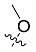	-H	32.00

**26**	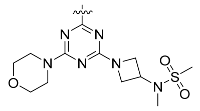	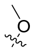	-H	6.00
**27**	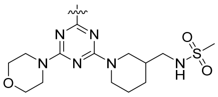	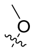	-H	5.80
**28**	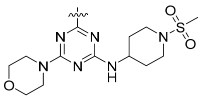	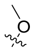	-H	14.00
**29**	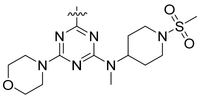	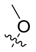	-H	2.30
**30**	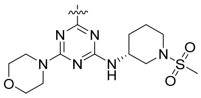	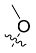	-H	25.00
**31**	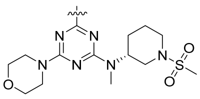	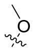	-H	20.00
**32**TS	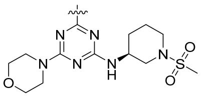	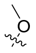	-H	27.00
**33**	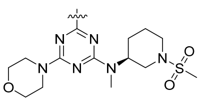	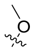	-H	14.00
**34**TS	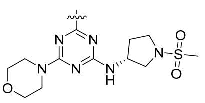	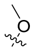	-H	11.00

**35**	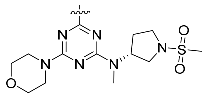	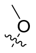	-H	7.50
**36**	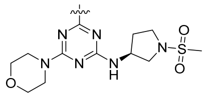	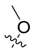	-H	7.80
**37**	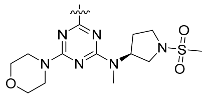	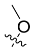	-H	3.80
**38**	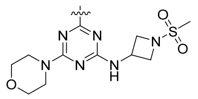	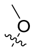	-H	4.40
**39**	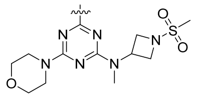	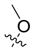	-H	4.90
**40**	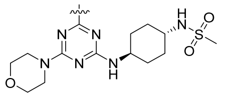	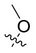	-H	6.10
**41**	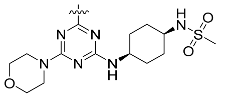	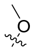	-H	8.00
**42**[TS]	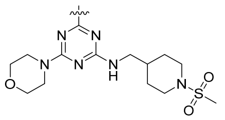	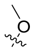	-H	3.50
**43**	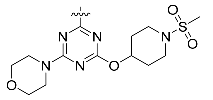	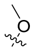	-H	32.00

**44**	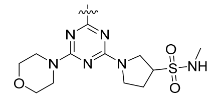	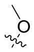	-H	6.10
**45**	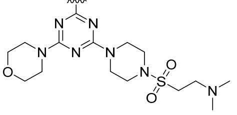	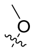	-H	5.00
**46**	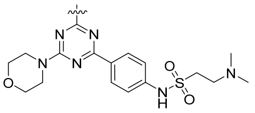	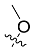	-H	6.00
**47**	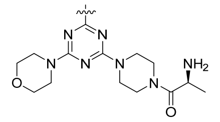	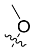	-H	77.00
***48**^{TS}	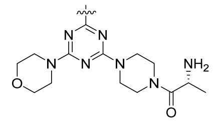	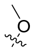	-H	106.00
**49**	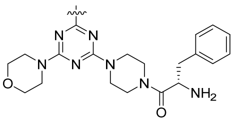	-H	-H	87.00
**50**	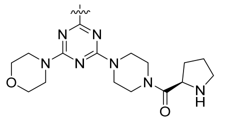	-H	-H	165.00
**51**	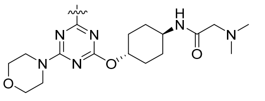	-H	-H	547.00
**52**	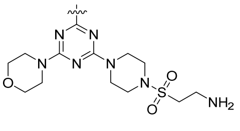	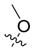	-H	5.40

**53**	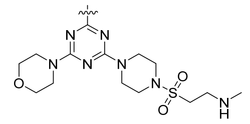	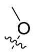	-H	6.50
**54**	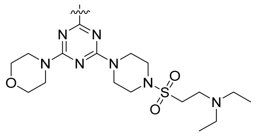	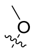	-H	5.90
**55**	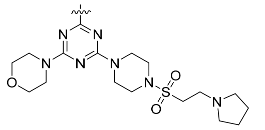	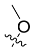	-H	14.00
**56**	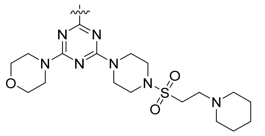	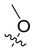	-H	6.00
**57**[TS]	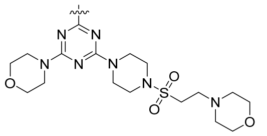	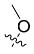	-H	18.00
**58**	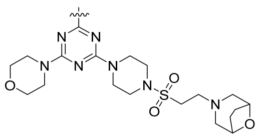	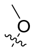	-H	12.00
**59**	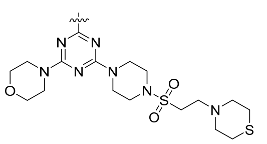	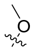	-H	5.00
**60**	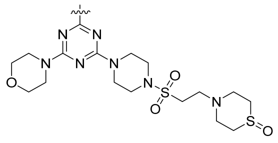	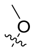	-H	8.90
**61**	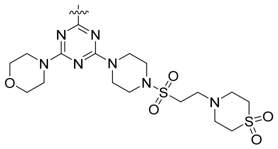	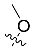	-H	7.50

**62**[TS]	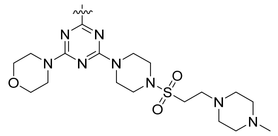	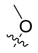	-H	13.00
**63**	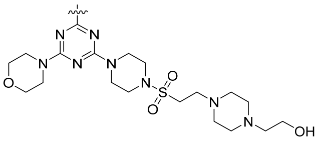	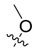	-H	2.30
**64**	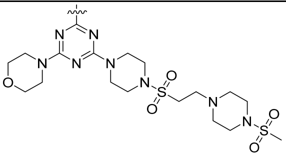	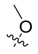	-H	7.80
**65**[TS]	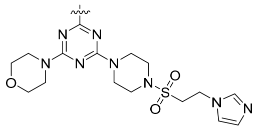	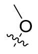	-H	5.80
**66**	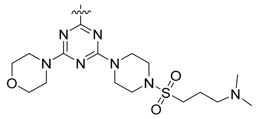	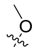	-H	7.90
**67**	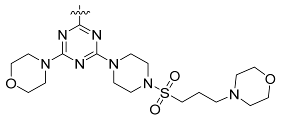	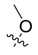	-H	5.40
**68**	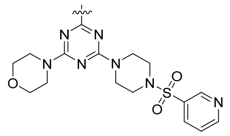	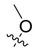	-H	5.80
**69**[TS]	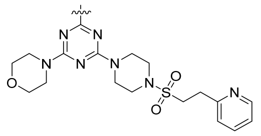	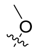	-H	2.70
**70**	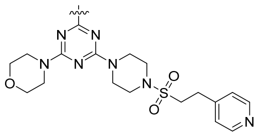	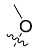	-H	5.30

**71**	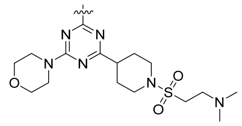	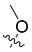	-H	2.40
**72**	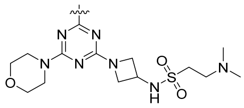	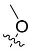	-H	13.00
**73**	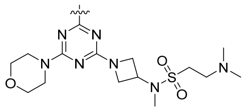	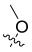	-H	46.00
**74**[TS]	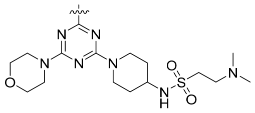	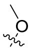	-H	7.30
**75**	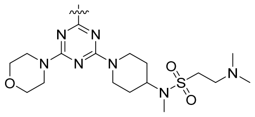	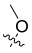	-H	68.00
**76**[TS]	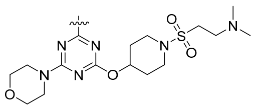	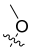	-H	7.30
**77**[TS]	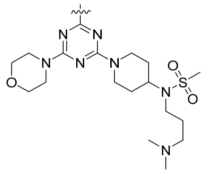	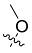	-H	55.00
**78**	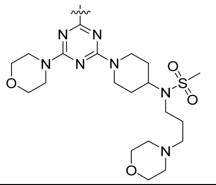	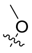	-H	24.00
**79**	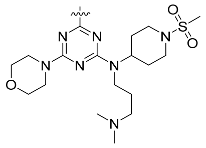	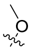	-H	29.00

**80**	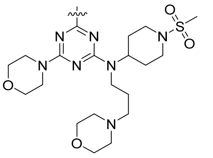	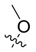	-H	20.00
**81**	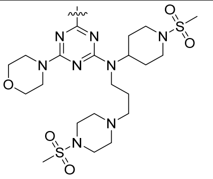	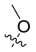	-H	23.00
**82**	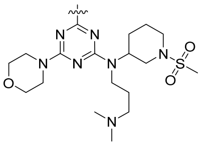	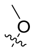	-H	70.00
**83**	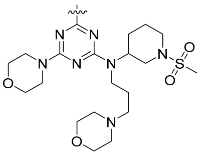	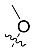	-H	72.00
**84**	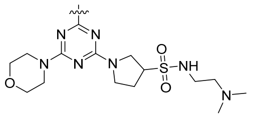	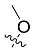	-H	9.10
**85**[TS]	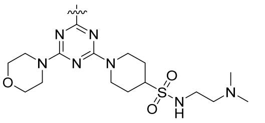	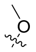	-H	27.00

TS = test set.

*Outlier molecule.

Table S2. The actual and predicted pIC_{50} values of all compounds

**Compd.**	**Actual**	**CoMFA**		**CoMSIA**	
		**Predicted**	**Residues**	**Predicted**	**Residues**
**1**	8.050	8.044	0.006	8.003	0.047
**2**	8.400	8.420	0.020	8.286	0.114
**3**	8.510	8.511	0.001	8.536	0.026
**4**	7.680	7.777	0.097	7.827	0.147
**5**	8.220	8.122	0.098	8.103	0.117
**6**	7.680	8.148	0.468	8.088	0.408
**7**	8.120	8.116	0.004	8.201	0.081
**8**	7.660	8.234	0.574	8.358	0.698
**9**	8.130	8.102	0.028	8.108	0.022
**10**	8.490	8.024	0.466	8.184	0.306
**11**	7.850	8.317	0.467	8.140	0.290

**12**	7.850	7.890	0.040	7.861	0.011
**13**	7.770	7.727	0.043	7.968	0.198
**14**	6.510	8.327	1.817	8.279	1.769
**15**	8.280	8.278	0.002	8.278	0.002
**16**	8.170	8.179	0.009	8.146	0.024
**17**	8.290	8.271	0.019	8.479	0.189
**18**	8.540	8.535	0.005	8.641	0.101
**19**	7.980	8.267	0.287	8.249	0.269
**20**	7.720	8.131	0.411	8.116	0.396
**21**	8.120	8.128	0.008	8.145	0.025
**22**	8.510	8.524	0.014	8.350	0.160
**23**	8.320	8.306	0.014	8.348	0.028
**24**	8.110	8.112	0.002	8.132	0.022
**25**	7.490	8.129	0.639	8.233	0.743
**26**	8.220	8.219	0.001	8.085	0.135
**27**	8.240	8.220	0.020	8.265	0.025
**28**	7.850	7.857	0.007	8.137	0.287
**29**	8.640	8.659	0.019	8.416	0.224
**30**	7.600	7.578	0.022	7.610	0.010
**31**	7.700	7.709	0.009	7.663	0.037
**32**	7.570	8.006	0.436	7.861	0.291
**33**	7.850	7.853	0.003	8.007	0.157
**34**	7.960	8.209	0.249	8.165	0.205
**35**	8.120	8.134	0.014	8.146	0.026
**36**	8.110	8.101	0.009	7.952	0.158
**37**	8.420	8.411	0.009	8.353	0.067
**38**	8.360	8.381	0.021	8.264	0.096
**39**	8.310	8.291	0.019	8.271	0.039
**40**	8.210	8.190	0.020	8.147	0.063
**41**	8.100	8.100	0.000	8.081	0.019
**42**	8.460	7.645	0.815	7.748	0.712
**43**	7.490	7.502	0.012	7.529	0.039
**44**	8.210	8.224	0.014	8.192	0.018
**45**	8.300	8.253	0.047	8.134	0.166
**46**	8.220	8.219	0.001	8.268	0.048
**47**	7.110	7.185	0.075	7.145	0.035
**48**	6.970	8.137	1.167	7.664	0.694
**49**	7.060	7.071	0.011	7.064	0.004
**50**	6.780	6.731	0.049	6.848	0.068
**51**	6.260	6.244	0.016	6.214	0.046
**52**	8.270	8.275	0.005	8.246	0.024
**53**	8.190	8.173	0.017	8.199	0.009
**54**	8.230	8.154	0.076	8.093	0.137

**55**	7.850	7.911	0.061	8.078	0.228
**56**	8.220	8.219	0.001	8.180	0.040
**57**	7.740	8.107	0.367	8.087	0.347
**58**	7.920	7.899	0.021	7.972	0.052
**59**	8.300	8.322	0.022	8.217	0.083
**60**	8.050	8.079	0.029	8.082	0.032
**61**	8.120	8.100	0.020	8.089	0.031
**62**	7.890	8.371	0.481	8.273	0.383
**63**	8.640	8.640	0.000	8.739	0.099
**64**	8.110	8.113	0.003	8.081	0.029
**65**	8.240	7.910	0.330	8.058	0.182
**66**	8.100	8.154	0.054	8.102	0.002
**67**	8.270	8.283	0.013	8.262	0.008
**68**	8.240	8.244	0.004	8.322	0.082
**69**	8.570	8.217	0.353	8.339	0.231
**70**	8.280	8.277	0.003	8.320	0.040
**71**	8.620	8.617	0.003	8.541	0.079
**72**	7.890	7.892	0.002	7.903	0.013
**73**	7.340	7.339	0.001	7.386	0.046
**74**	8.140	7.761	0.379	7.879	0.261
**75**	7.170	7.156	0.014	7.211	0.041
**76**	8.140	7.708	0.432	7.507	0.633
**77**	7.260	7.664	0.404	7.671	0.411
**78**	7.620	7.616	0.004	7.579	0.041
**79**	7.540	7.562	0.022	7.614	0.074
**80**	7.700	7.713	0.013	7.609	0.091
**81**	7.640	7.644	0.004	7.596	0.044
**82**	7.150	7.135	0.015	7.106	0.044
**83**	7.140	7.143	0.003	7.078	0.062
**84**	8.040	7.581	0.459	8.011	0.029
**85**	7.570	7.568	0.002	7.590	0.020

^{1}H NMR, ^{19}FNMR, ^{13}CNMR and HRMS spectra of the important compounds:

2-ethoxy-6-nitroaniline (92b)

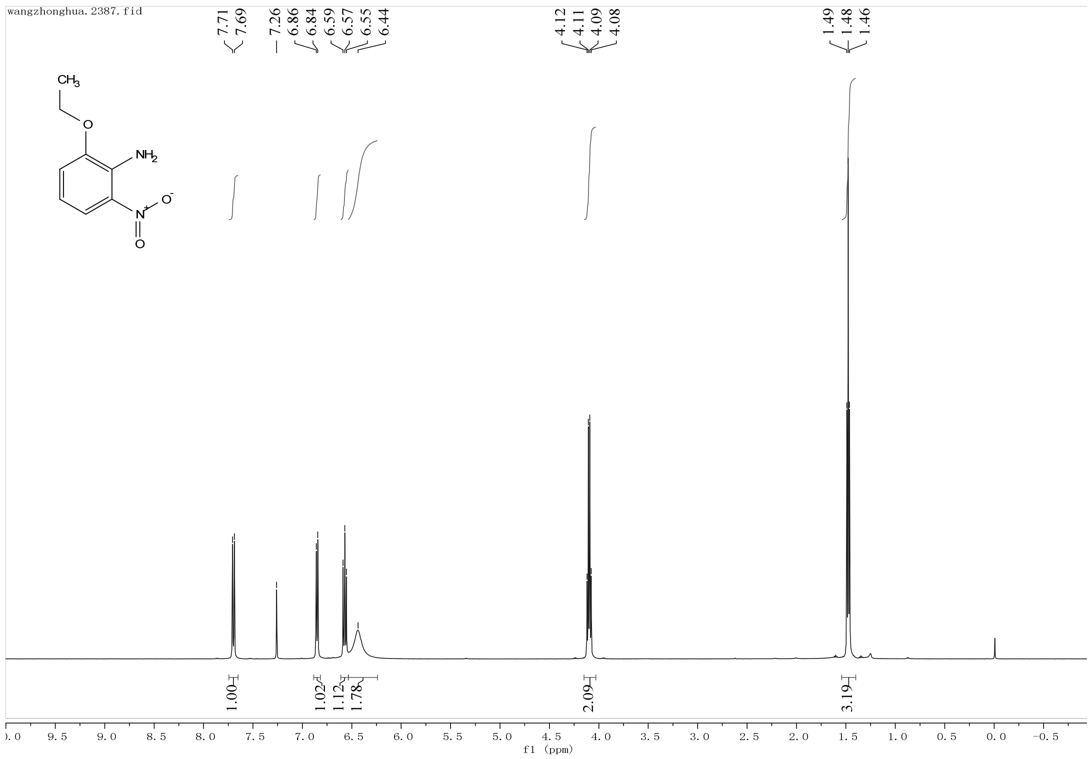

4-methoxy-2-(trifluoromethyl)-1H-benzo[d]imidazole(93a)

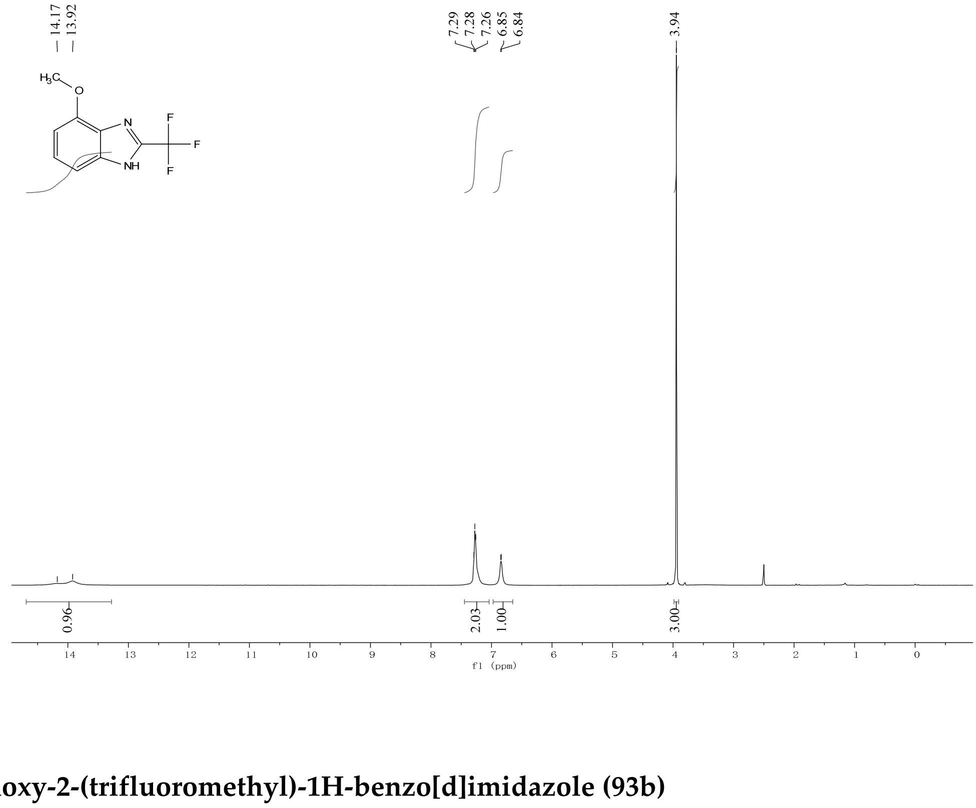

4-ethoxy-2-(trifluoromethyl)-1H-benzo[d]imidazole (93b)

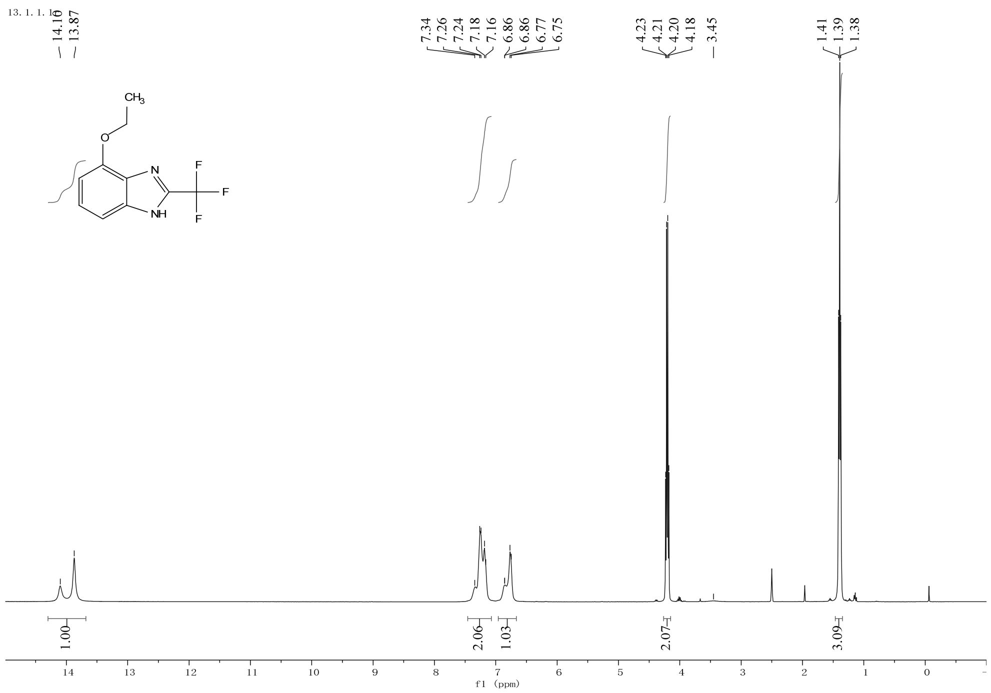
13.1.1.1ō
14.10
13.87
7.34
7.26
7.24
7.18
7.16
6.86
6.86
6.77
6.75
4.23
4.21
4.20
4.18
3.45
1.41
1.39
1.38
CH3
O
N
NH
F
F
F
1.00
2.06
1.03
2.07
3.09
14 13 12 11 10 9 8 7 6 5 4 3 2 1 0
f1 (ppm)

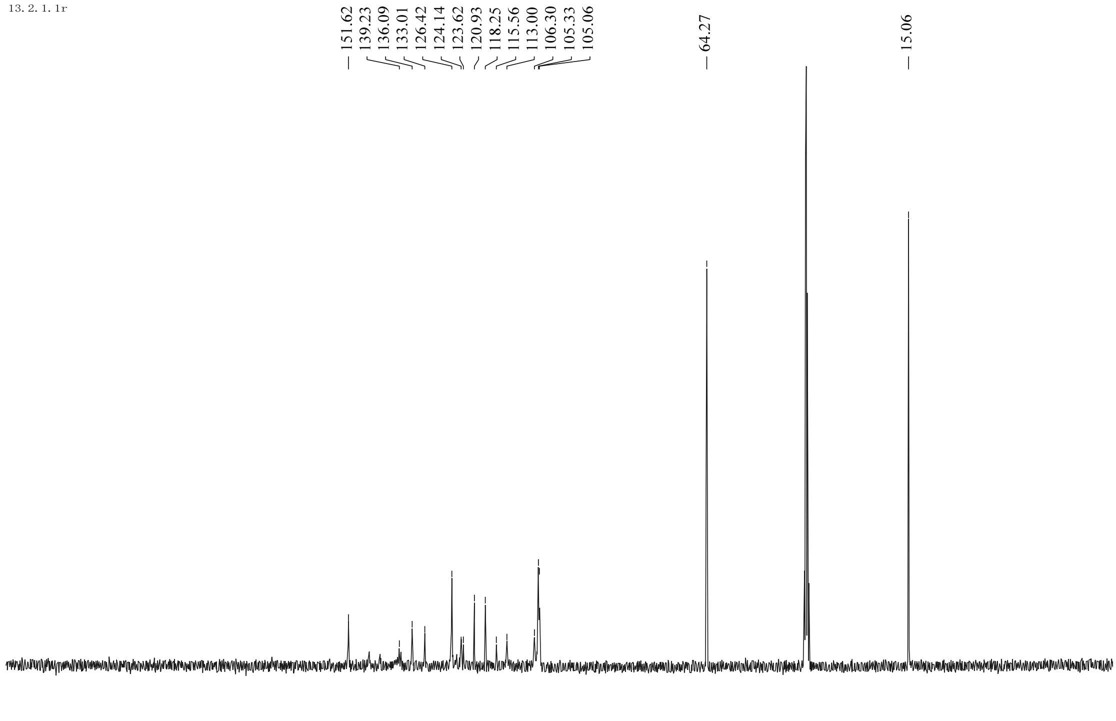
13.2.1.1r
151.62
139.23
136.09
133.01
126.42
124.14
123.62
120.93
118.25
115.56
113.00
106.30
105.33
105.06
64.27
15.06
230 220 210 200 190 180 170 160 150 140 130 120 110 100 90 80 70 60 50 40 30 20 10 0 −10 −20 −30
f1 (ppm)

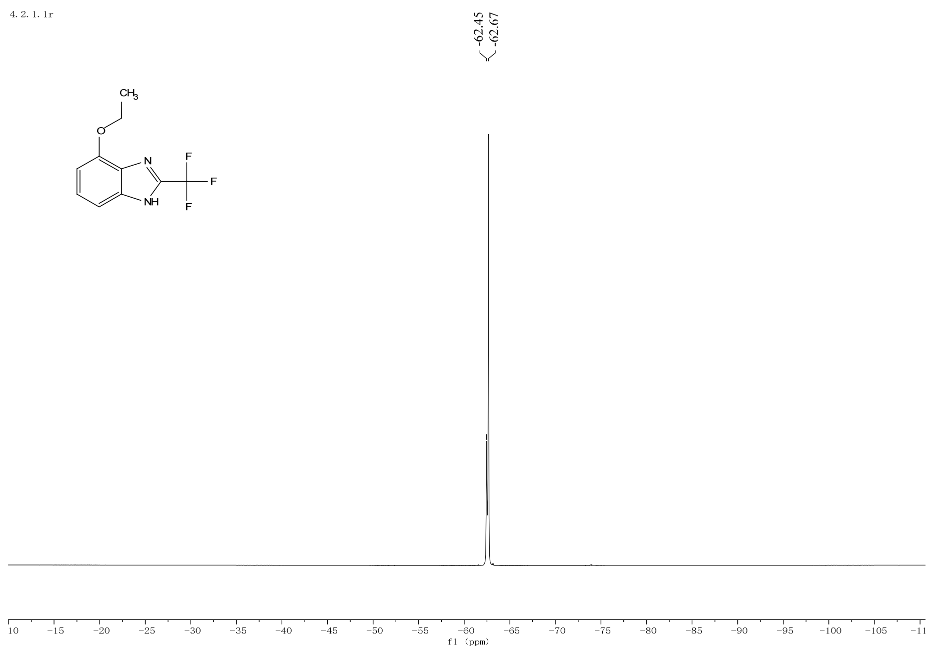

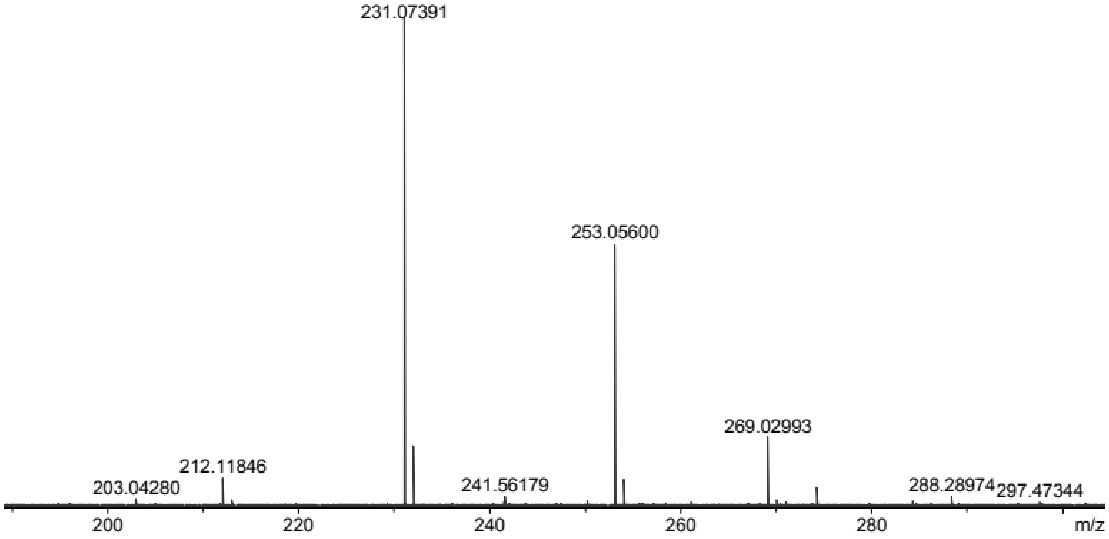

4-(4-chloro-6-(4-methoxy-2-(trifluoromethyl)-1H-benzo[d]imidazol-1-yl)-1,3,5-triazin-2-yl)morpholine (95a)

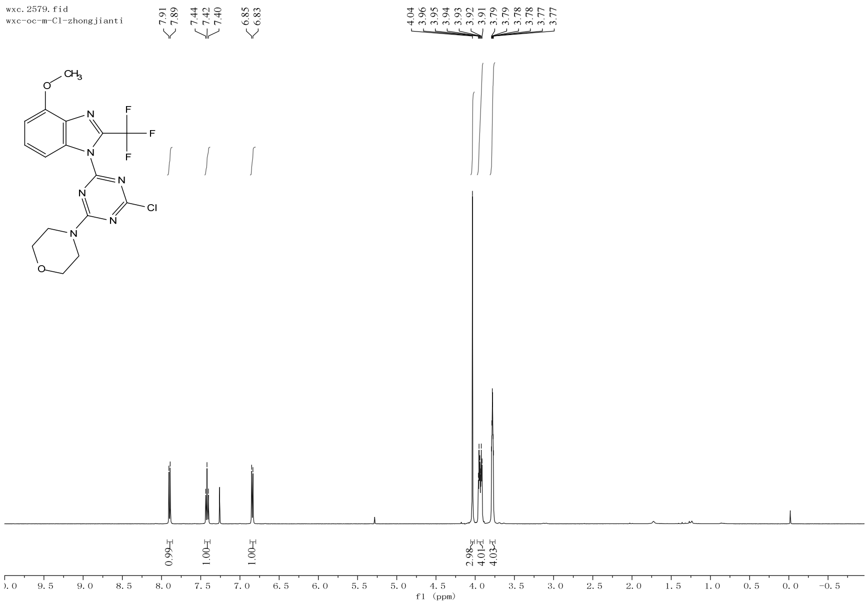
wxc.2579.fid
wxc-oc-m-Cl-zhongjianti
7.91
7.89
7.44
7.42
7.40
6.85
6.83
4.04
3.96
3.95
3.94
3.93
3.92
3.91
3.79
3.79
3.78
3.78
3.77
3.77
0.99
1.00
1.00
2.98
4.01
4.03
f1 (ppm)

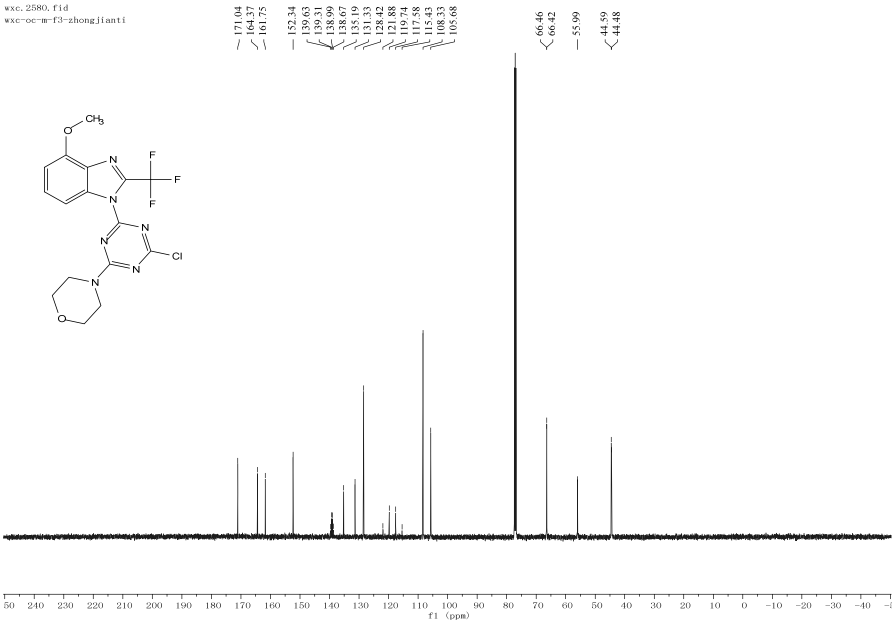
wxc.2580.fid
wxc-oc-m-f3-zhongjianti
171.04
164.37
161.75
152.34
139.63
139.31
138.99
138.67
135.19
131.33
128.42
121.88
119.74
117.58
115.43
108.33
105.68
66.46
66.42
55.99
44.59
44.48
f1 (ppm)

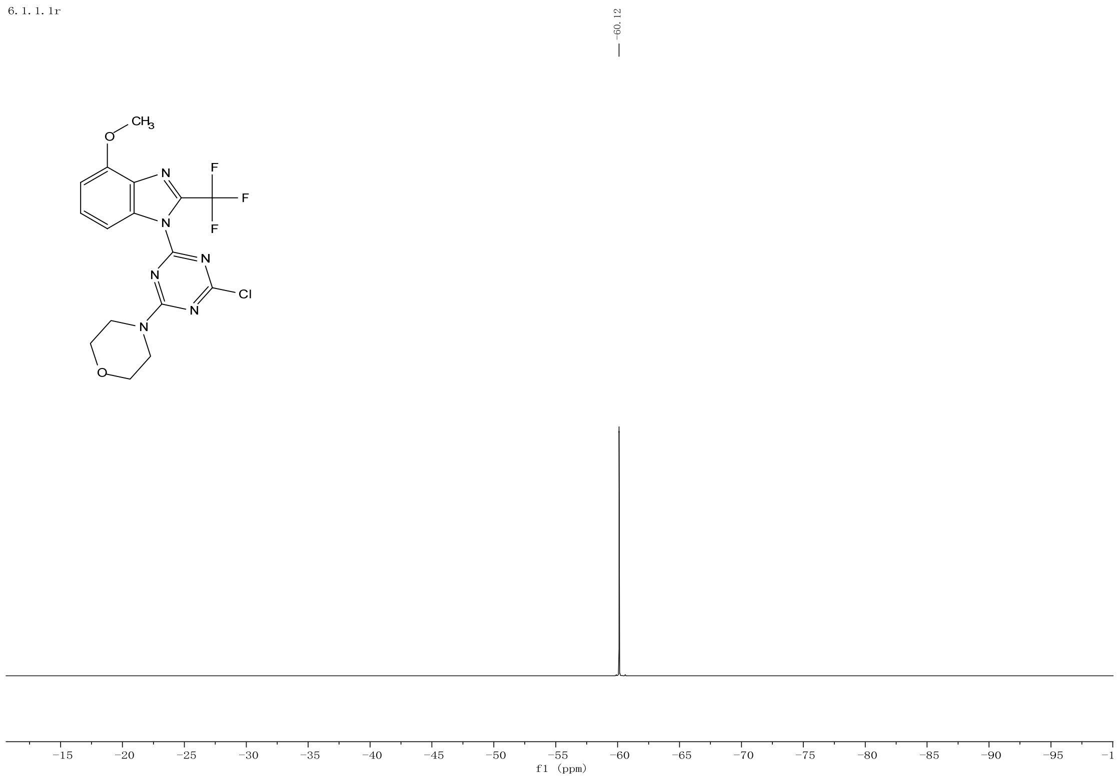

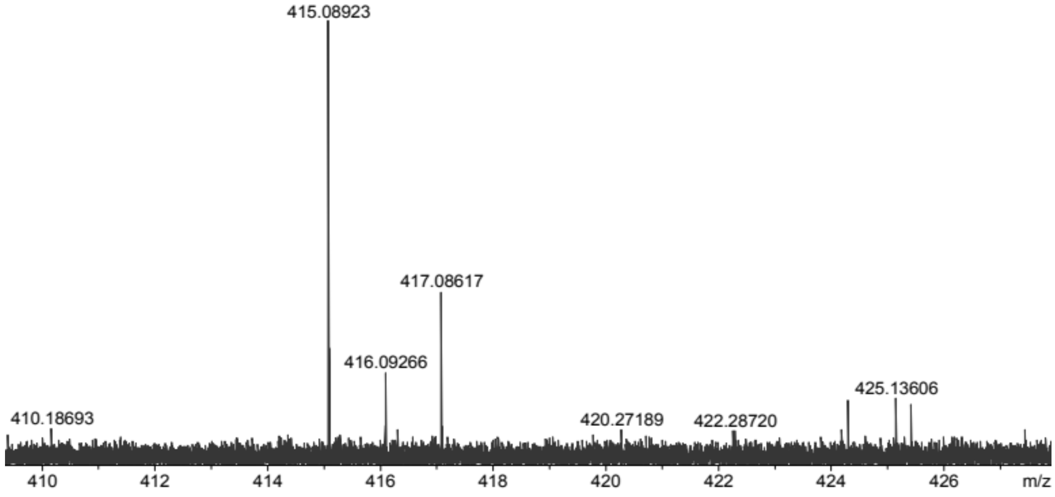

4-(4-chloro-6-(4-ethoxy-2-(trifluoromethyl)-1H-benzo[d]imidazol-1-yl)-1,3,5-triazin-2-yl)morpholine (95b)

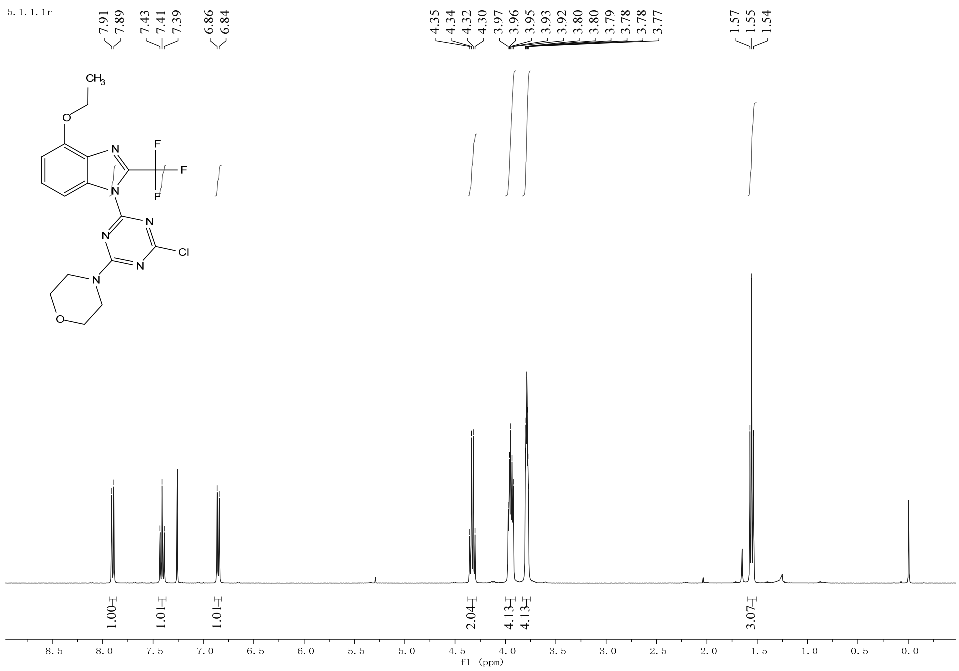
5.1.1.1r
7.91
7.89
7.43
7.41
7.39
6.86
6.84
4.35
4.34
4.32
4.30
3.97
3.96
3.95
3.93
3.92
3.80
3.80
3.79
3.78
3.78
3.77
1.57
1.55
1.54
CH3
O
N
F
F
F
N
N
N
N
Cl
N
O
1.00
1.01
1.01
2.04
4.13
4.13
3.07
f1 (ppm)

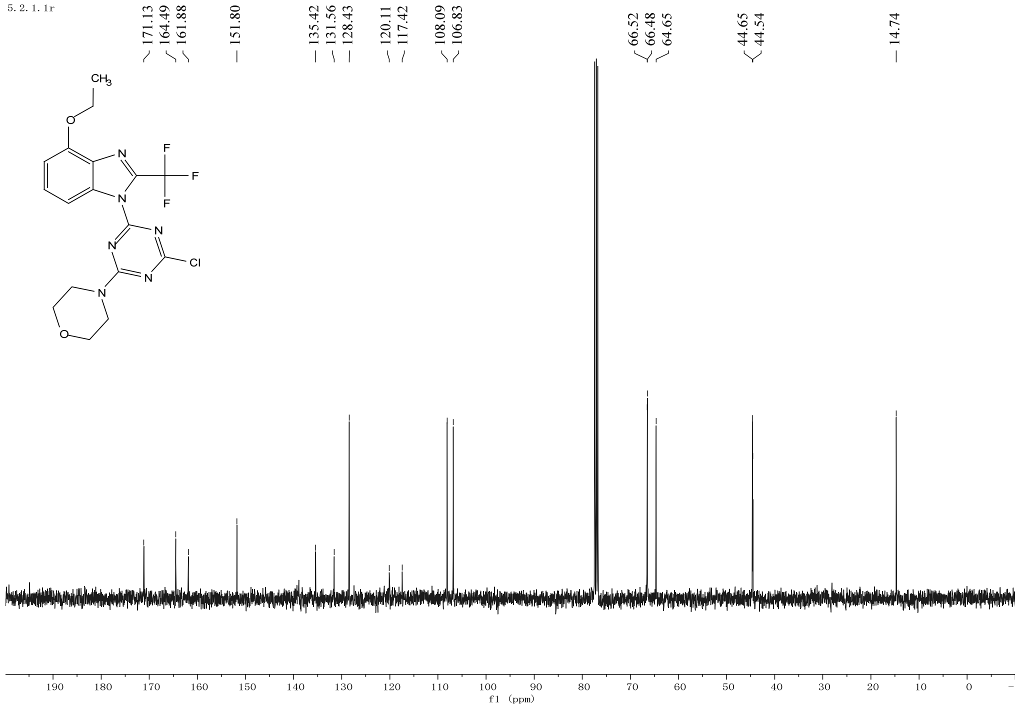
5.2.1.1r
171.13
164.49
161.88
151.80
135.42
131.56
128.43
120.11
117.42
108.09
106.83
66.52
66.48
64.65
44.65
44.54
14.74
CH3
O
N
F
F
F
N
N
N
N
Cl
N
O
f1 (ppm)

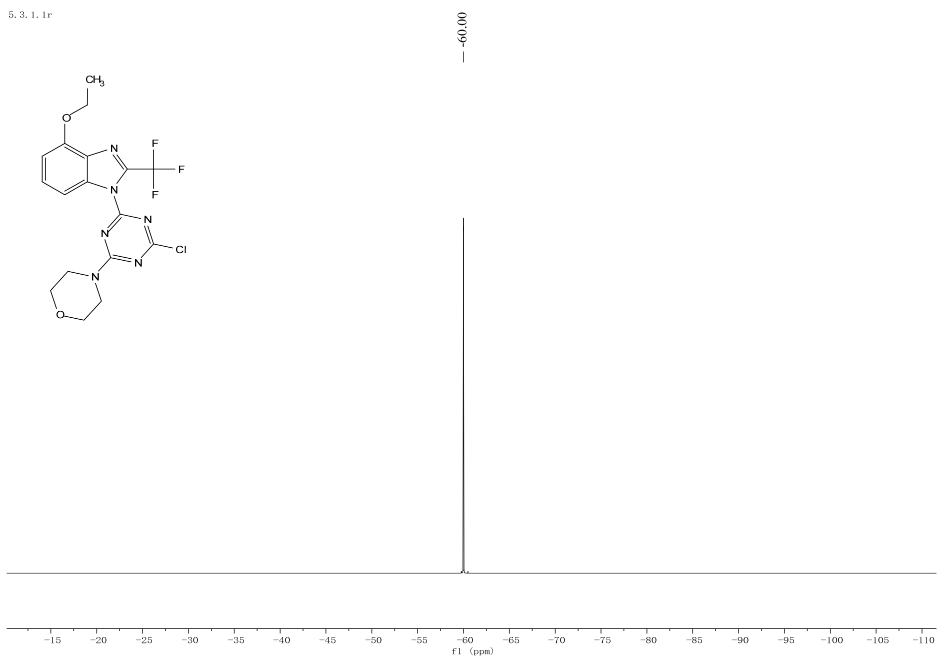

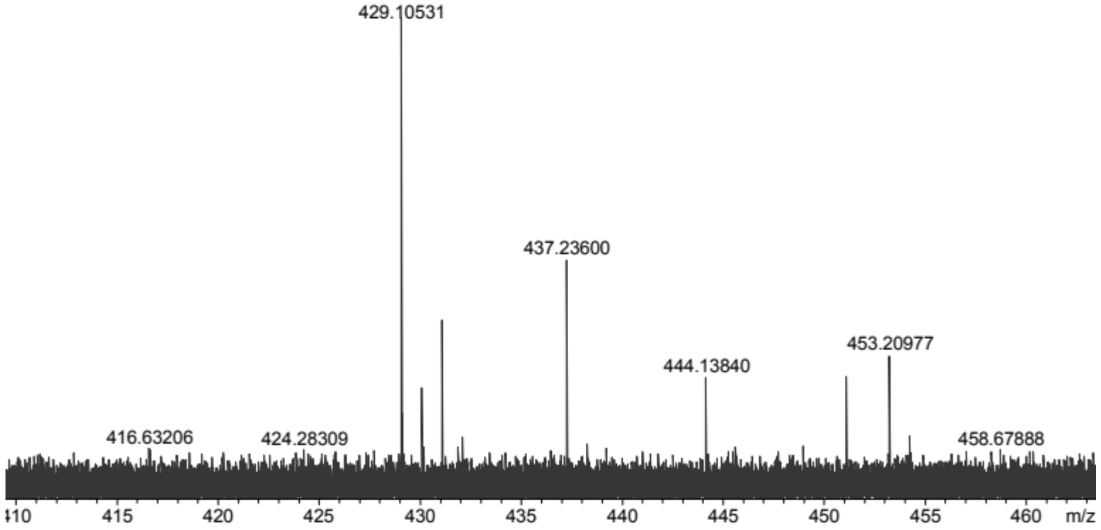

4-(4-methoxy-2-(trifluoromethyl)-1H-benzo[d]imidazol-1-yl)-N-methyl-N-(1-(methylsulfonyl)piperidin-4-yl)-6-morpholino-1,3,5-triazin-2-amine (86)

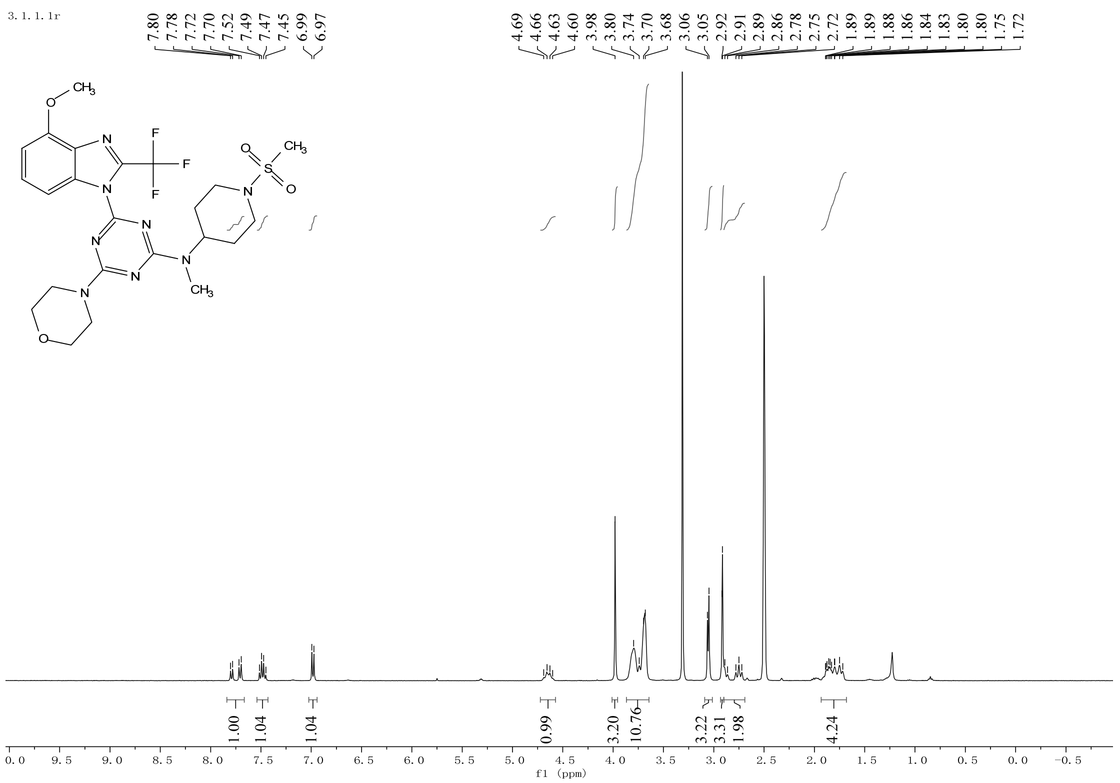
3.1.1.1r
7.80 7.78 7.72 7.70 7.52 7.49 7.47 7.45 6.99 6.97
4.69 4.66 4.63 4.60 3.98 3.80 3.74 3.70 3.68 3.06 3.05 2.92 2.91 2.89 2.86 2.78 2.75 2.72 1.89 1.89 1.88 1.86 1.84 1.83 1.80 1.80 1.75 1.72
1.00 1.04 1.04 0.99 3.20 10.76 3.22 3.31 1.98 4.24
f1 (ppm)

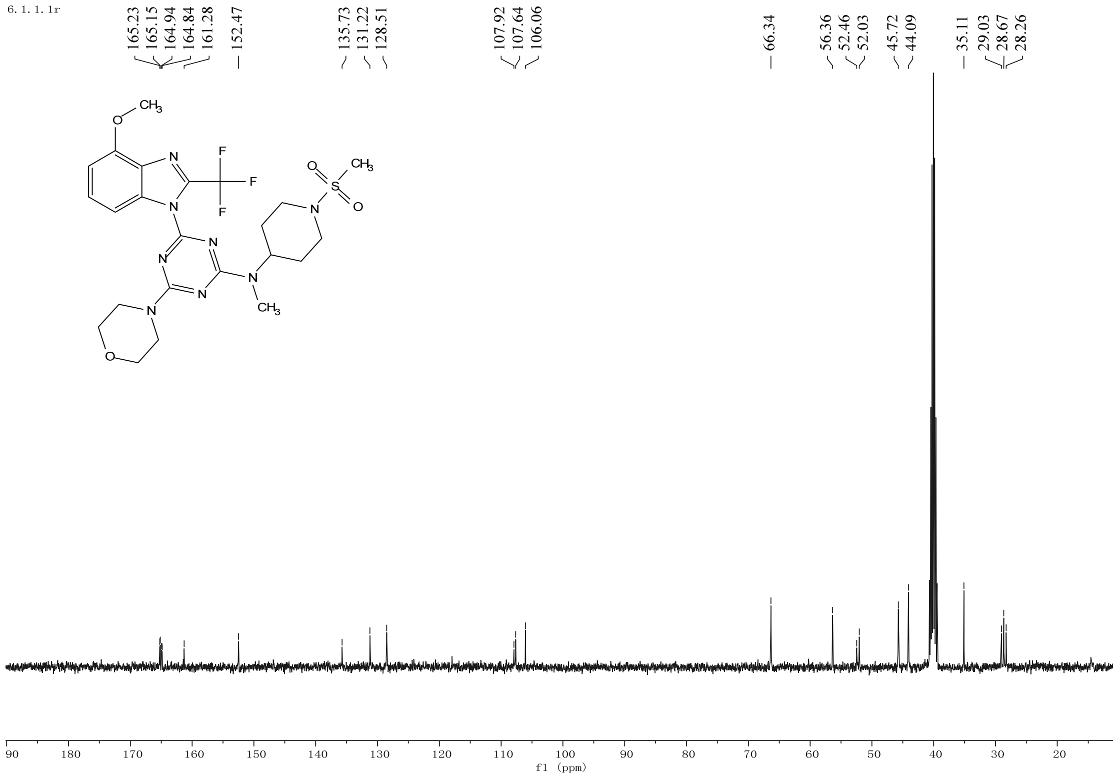
6.1.1.1r
165.23 165.15 164.94 164.84 161.28 152.47 135.73 131.22 128.51 107.92 107.64 106.06 66.34 56.36 52.46 52.03 45.72 44.09 35.11 29.03 28.67 28.26
f1 (ppm)

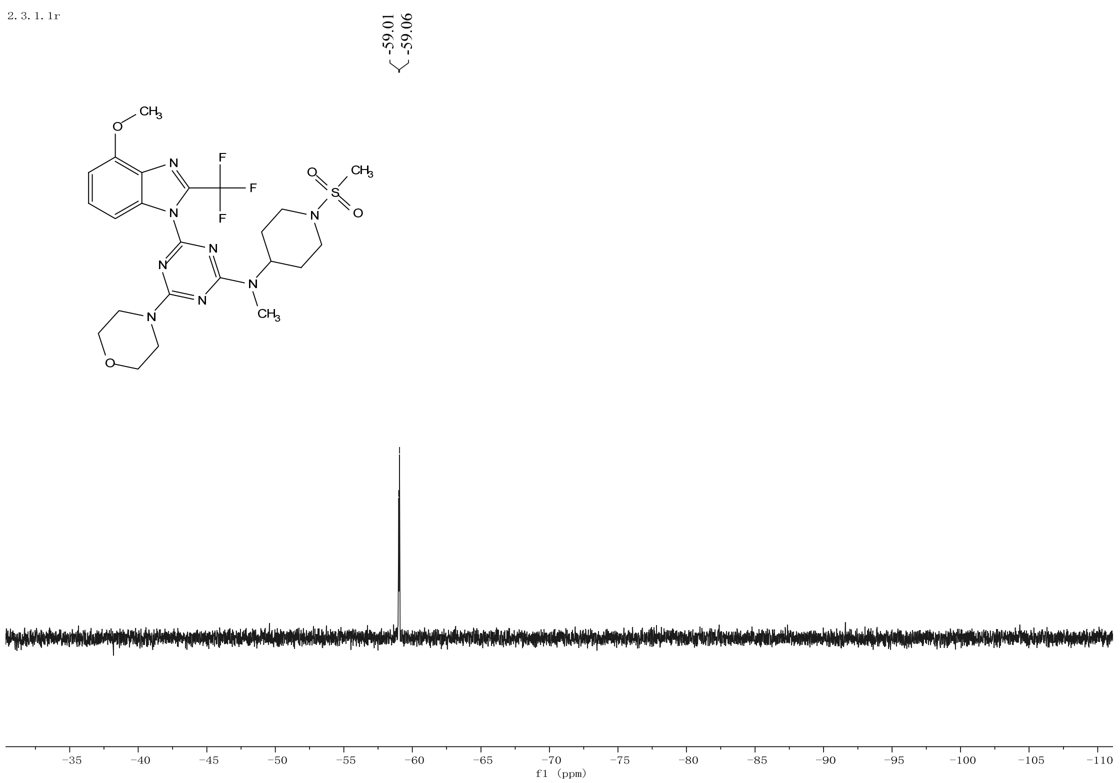

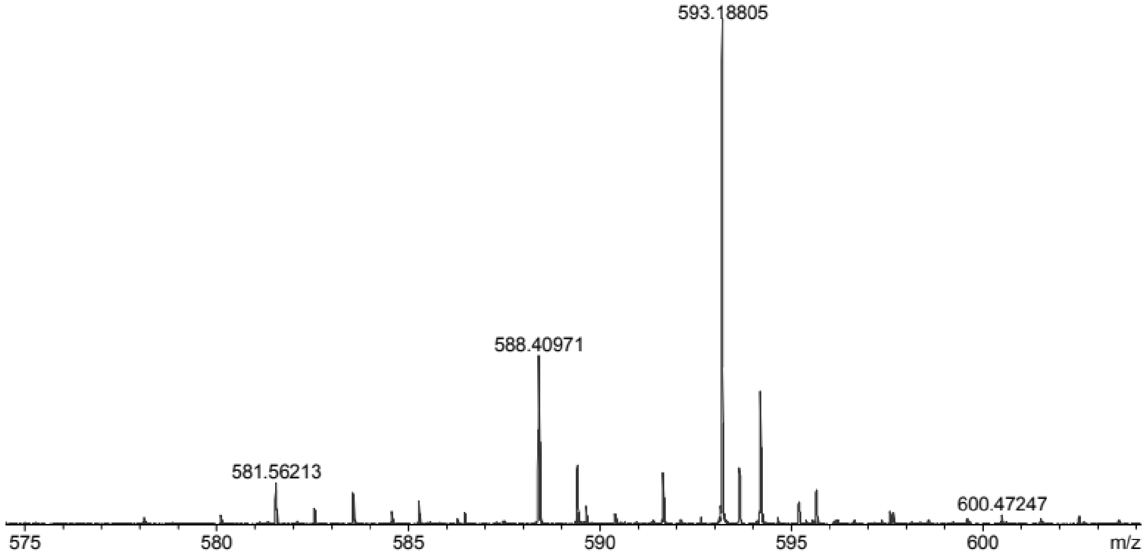

4-(4-ethoxy-2-(trifluoromethyl)-1H-benzo[d]imidazol-1-yl)-N-methyl-N-(1-(methylsulfonyl)piperidin-4-yl)-6-morpholino-1,3,5-triazin-2-amine (87)

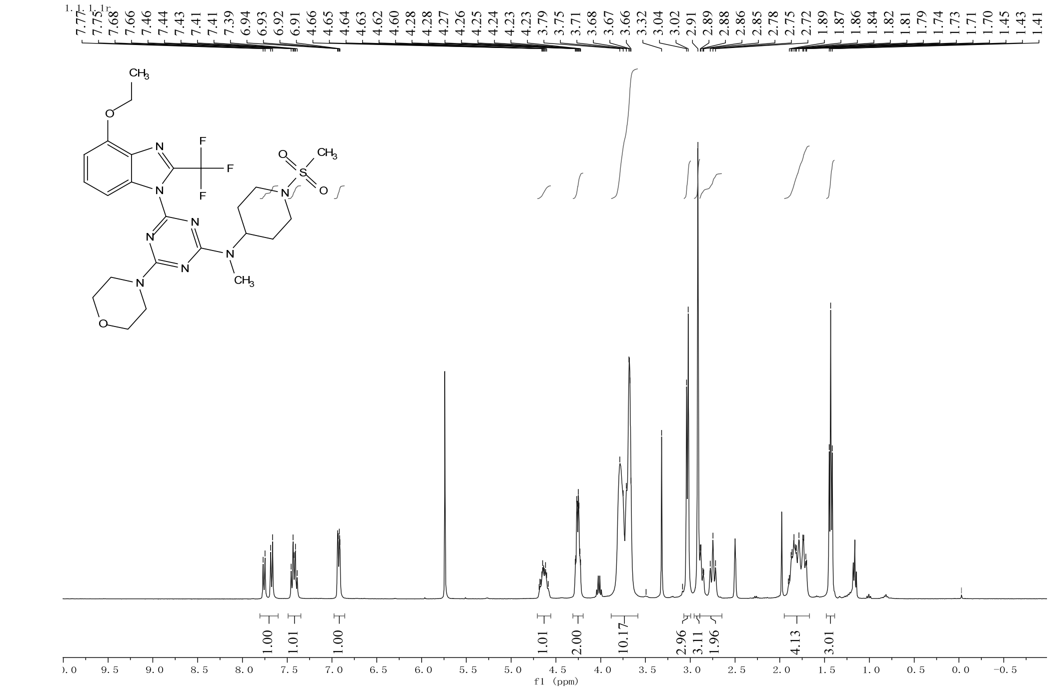
7.77
7.75
7.68
7.66
7.46
7.44
7.43
7.41
7.41
7.39
6.94
6.93
6.92
6.91
4.66
4.65
4.64
4.63
4.62
4.60
4.28
4.28
4.27
4.26
4.25
4.24
4.23
4.23
3.79
3.75
3.71
3.68
3.67
3.66
3.32
3.04
3.02
2.91
2.89
2.88
2.86
2.85
2.78
2.75
2.72
1.89
1.87
1.86
1.84
1.82
1.81
1.79
1.74
1.73
1.71
1.70
1.45
1.43
1.41
1.00
1.01
1.00
1.01
2.00
10.17
2.96
3.11
1.96
4.13
3.01
f1 (ppm)

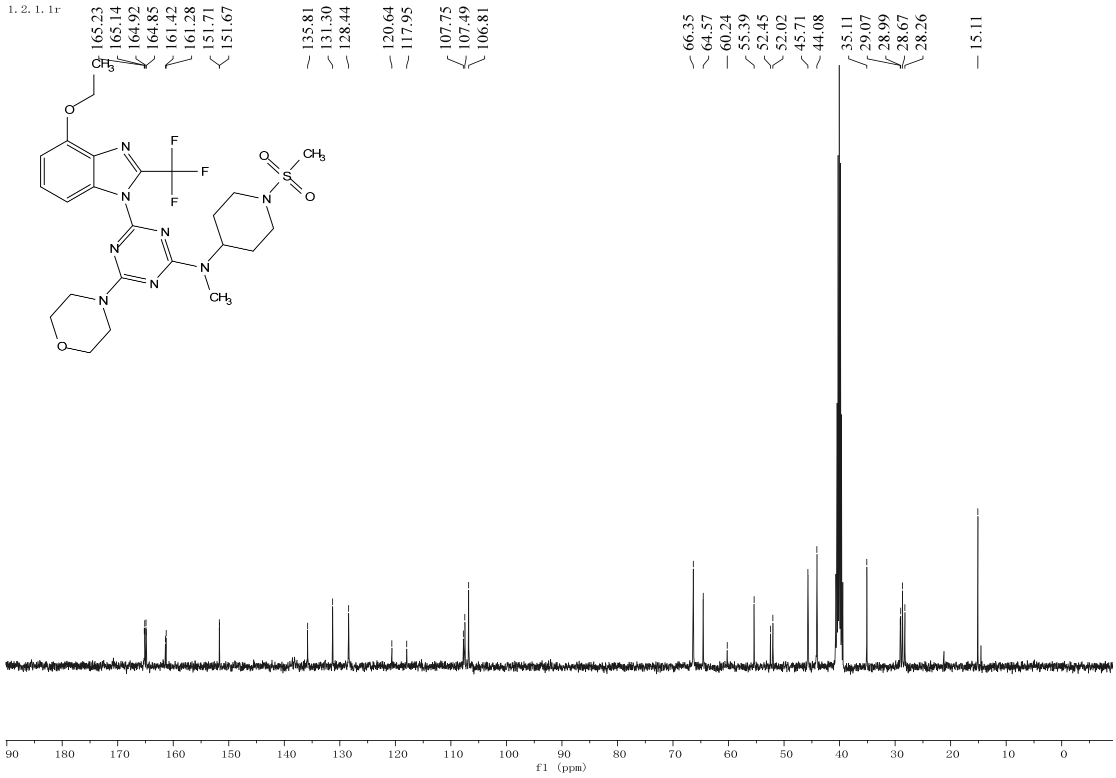
165.23
165.14
164.92
164.85
161.42
161.28
151.71
151.67
135.81
131.30
128.44
120.64
117.95
107.75
107.49
106.81
66.35
64.57
60.24
55.39
52.45
52.02
45.71
44.08
35.11
29.07
28.99
28.67
28.26
15.11
f1 (ppm)

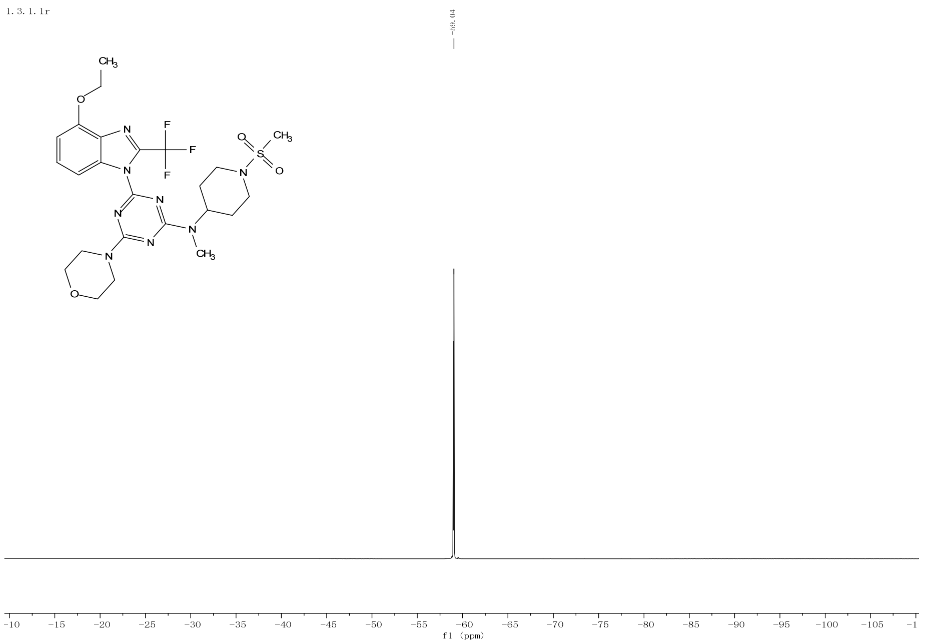
1.3.1.1r
-59.04
f1 (ppm)

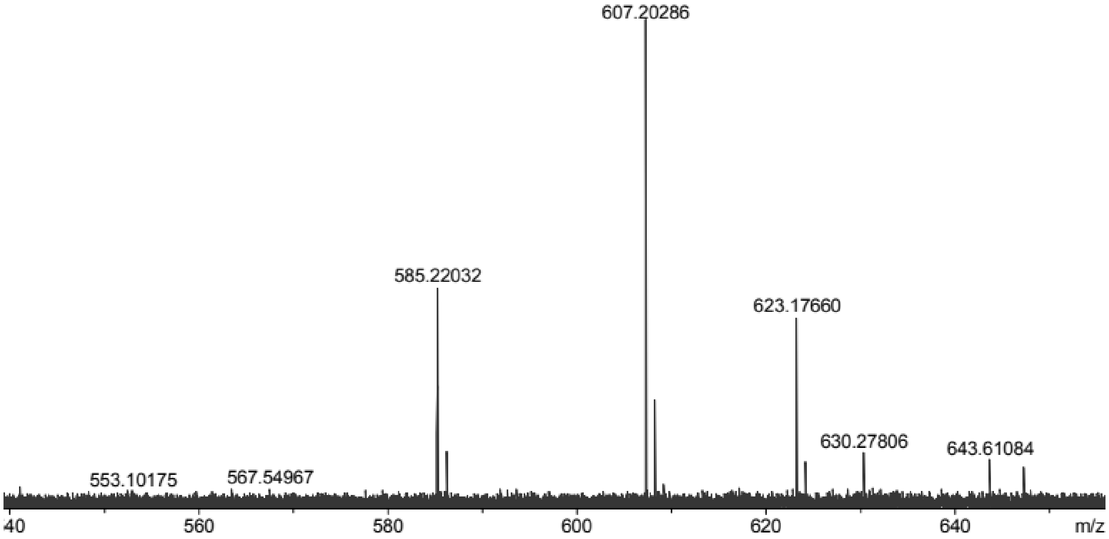
607.20286
585.22032
623.17660
630.27806
643.61084
553.10175
567.54967
m/z
